# hnRNP R negatively regulates transcription by modulating the association of P‐TEFb with 7SK and BRD4

**DOI:** 10.15252/embr.202255432

**Published:** 2022-07-20

**Authors:** Changhe Ji, Chunchu Deng, Katharina Antor, Thorsten Bischler, Cornelius Schneider, Utz Fischer, Michael Sendtner, Michael Briese

**Affiliations:** ^1^ Institute of Clinical Neurobiology University Hospital Wuerzburg Wuerzburg Germany; ^2^ Core Unit Systems Medicine University of Wuerzburg Wuerzburg Germany; ^3^ Department of Biochemistry, Theodor Boveri Institute University of Wuerzburg Wuerzburg Germany

**Keywords:** 7SK, BRD4, hnRNP R, P‐TEFb, transcription, History & Philosophy of Science

## Abstract

The P‐TEFb complex promotes transcription elongation by releasing paused RNA polymerase II. P‐TEFb itself is known to be inactivated through binding to the non‐coding RNA 7SK but there is only limited information about mechanisms regulating their association. Here, we show that cells deficient in the RNA‐binding protein hnRNP R, a known 7SK interactor, exhibit increased transcription due to phosphorylation of RNA polymerase II. Intriguingly, loss of hnRNP R promotes the release of P‐TEFb from 7SK, accompanied by enhanced hnRNP A1 binding to 7SK. Additionally, we found that hnRNP R interacts with BRD4, and that hnRNP R depletion increases BRD4 binding to the P‐TEFb component CDK9. Finally, CDK9 is stabilized upon loss of hnRNP R and its association with Cyclin K is enhanced. Together, our results indicate that hnRNP R negatively regulates transcription by modulating the activity and stability of the P‐TEFb complex, exemplifying the multimodal regulation of P‐TEFb by an RNA‐binding protein.

## Graphical abstract

The RNA‐binding protein hnRNP R inhibits P‐TEFb‐mediated phosphorylation of RNA polymerase II, thereby modulating transcription. P‐TEFb remodelling and release from 7SK in an hnRNP R‐dependent manner contribute to this effect.

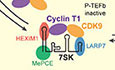

## Introduction

Gene expression is tightly regulated to facilitate transcript production based on cellular requirements. The non‐coding RNA 7SK controls transcription by regulating the kinase activity of the positive transcription elongation factor b (P‐TEFb) complex composed of cyclin‐dependent kinase 9 (CDK9) and Cyclin T1 (Nguyen *et al*, 2001; Yang *et al*, 2001). 7SK is highly structured containing four stem loops. It is protected from exonucleolytic degradation by binding of methyl phosphate capping enzyme (MePCE) to its 5′ end and of La‐related protein 7 (LARP7) to its 3′ end (Krueger *et al*, 2008; Markert *et al*, 2008; Xue *et al*, 2010; Muniz *et al*, 2013). This 7SK/MePCE/LARP7 ‘core’ ribonucleoprotein particle (RNP) serves as a scaffold to modulate the activity of regulatory protein complexes in the nucleus but also in the cytosol.

During transcription, RNA polymerase II (RNA pol II) tends to pause downstream of transcription start sites of most genes (Noe Gonzalez *et al*, 2021). Release of paused RNA pol II requires the activity of the P‐TEFb complex, which phosphorylates the C‐terminal domain of RNA pol II at Serine 2 (Ser2) as well as the negative transcription elongation factors DRB sensitivity‐inducing factor (DSIF) and negative elongation factor (NELF), allowing processive elongation to occur (Quaresma *et al*, 2016). P‐TEFb itself can be sequestered by 7SK through interaction with hexamethylene bisacetamide‐induced protein 1 (HEXIM1) bound to stem‐loop 1 (Yik *et al*, 2003; Egloff *et al*, 2006). The inhibition of P‐TEFb by 7SK serves to fine‐tune RNA pol II pausing: high levels of RNA pol II pausing induce P‐TEFb release from 7SK to facilitate transcriptional re‐activation and vice versa. The mechanism behind the 7SK‐mediated control of P‐TEFb has been pinpointed through the identification of heterogeneous nuclear ribonucleoproteins (hnRNPs), among them hnRNP A1 and R, as 7SK interactors by proteomics approaches (Barrandon *et al*, 2007; Van Herreweghe *et al*, 2007; Ji *et al*, 2021). 7SK has also been identified as the major RNA target of hnRNP R by individual‐nucleotide resolution crosslinking and immunoprecipitation (iCLIP; Briese *et al*, 2018). Whereas P‐TEFb binding to 7SK requires stem‐loop 1 and 4 (Egloff *et al*, 2006), hnRNPs interact with stem‐loop 3 of 7SK (Van Herreweghe *et al*, 2007; Briese *et al*, 2018). Importantly, binding of P‐TEFb and hnRNPs to 7SK is mutually exclusive and can be shifted towards one or the other depending on the transcriptional requirements (Briese & Sendtner, 2021). When nascent RNA levels are lowered by transcriptional inhibition, hnRNPs binding to 7SK is enhanced, causing the release of P‐TEFb from 7SK for transcription re‐activation. Conversely, increased production of nascent RNA is thought to induce re‐distribution of hnRNPs from 7SK towards newly synthesized RNA, thereby facilitating P‐TEFb sequestration by 7SK. Thus, the balance between 7SK/P‐TEFb and 7SK/hnRNP complexes represents a regulatory mechanism to re‐adjust transcription globally. Nevertheless, while this model has been supported by knockdown approaches showing that depletion of hnRNPs shifts 7SK binding towards P‐TEFb, rigorous testing of this hypothesis using cells with chronic depletion of one of the 7SK‐interacting hnRNPs has been lacking.

Here, we investigated the consequences of hnRNP R depletion for transcription using hnRNP R knockout cells. We observed that, contrary to the canonical model of 7SK function, transcription was enhanced in hnRNP R‐deficient cells accompanied by increased Ser2 phosphorylation of RNA pol II. Mechanistically, loss of hnRNP R led to the release of P‐TEFb from 7SK through the enhanced association of hnRNP A1 with 7SK. Additionally, the interaction of CDK9 with bromodomain‐containing protein 4 (BRD4), a positive regulator of P‐TEFb function, was increased by hnRNP R depletion. Together, our findings extend the current model of 7SK‐mediated P‐TEFb regulation by showing that the balance between different 7SK/hnRNP subcomplexes determines the availability of 7SK for P‐TEFb sequestration.

## Results

### Loss of hnRNP R activates transcription

**Figure 1:**
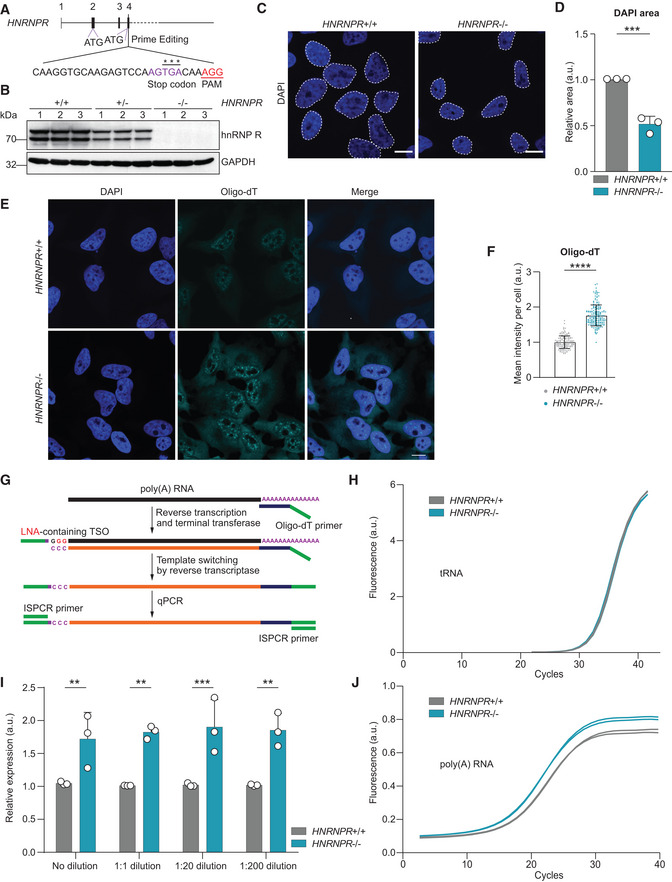
hnRNP R depletion increases transcription ASchematic of the strategy for the generation of hnRNP R knockout cells by prime editing. The PAM sequence is underlined in red, the protospacer sequence recognized by the pegRNA is marked in black, the inserted nucleotides are marked in purple and the TGA stop codon is indicated by asterisks.BWestern blot analysis of hnRNP R in three independent *HNRNPR* wildtype (+/+), heterozygous (+/−) and homozygous (−/−) HeLa cell lines.CConfocal microscopy images of nuclei stained with DAPI. Scale bar: 10 μm.DQuantification of the DAPI area in (C). Data are mean with standard deviation (SD); ***P ≤ 0.001; unpaired two‐tailed *t*‐test (*n* = 3 biological replicates).ERNA FISH with a Cy3‐labeled oligo‐dT probe. DAPI was used for nuclear staining. Scale bar: 10 μm.FQuantification of mean fluorescence intensity of the Cy3 oligo‐dT signal in (E). Data are mean with SD; ****P ≤ 0.0001; Mann–Whitney test (*n* = 129 cells for *HNRNPR* ^+/+^ and *n* = 176 cells for *HNRNPR* ^−/−^).GSchematic of the mRNA quantification procedure (Picelli *et al*, 2014). TSO, template‐switching oligonucleotide.HFluorescence detection of tRNA amplification by qPCR.IQuantification of relative mRNA levels detected by qPCR from cDNA at different dilutions. Data are mean with SD; **P ≤ 0.01, ***P ≤ 0.001; two‐way ANOVA with Sidak's multiple comparisons test (*n* = 3 biological replicates).JFluorescence detection of mRNA amplification by qPCR using ISPCR primers.

To investigate the functions of hnRNP R in transcriptional regulation, we knocked out hnRNP R in HeLa cells by prime editing (Anzalone *et al*, 2019). Alternative splicing of exon 2 generates two *HNRNPR* mRNA isoforms with a start codon in either exon 2 or exon 4, giving rise to two hnRNP R protein isoforms that differ at the N‐terminus (Ghanawi *et al*, 2021). To abolish production of both isoforms, we inserted an in‐frame stop codon in exon 4 of *HNRNPR* downstream of the start codon of the short hnRNP R isoform (Fig 1A; Appendix Fig S1A–G). In homozygous *HNRNPR* ^−/−^ cells, hnRNP R protein was undetectable, and in +/− cells, both hnRNP R isoforms were reduced to ∼ 35% (Fig 1B; Appendix Fig S1H). Levels of 7SK itself were unaffected by hnRNP R depletion (Appendix Fig S1H). Also, levels of 7SK interactors (MePCE, LARP7, HEXIM1, hnRNP A1), components of messenger ribonucleoprotein particles (PABPC1, TIAR, TDP‐43), ribosomal proteins (RPS5) or the spliceosomal machinery (SMN, SmB/B′) were unchanged in *HNRNPR* ^−/−^ cells (Appendix Fig S2A–L).

Compared to wild‐type cells, nuclei of *HNRNPR* ^−/−^ cells were smaller (Fig 1C and D) and their proliferation was enhanced (Appendix Fig S3A–C), which prompted us to investigate their transcription. To do so, we first assessed their total mRNA levels by RNA fluorescent *in situ* hybridization (FISH) with a fluorescently labelled oligo‐dT probe. This revealed increased mRNA levels in *HNRNPR* ^−/−^ compared to +/+ cells (Fig 1E and F). To confirm this finding, we reverse‐transcribed mRNA with an oligo‐dT‐containing primer followed by template switching (Picelli *et al*, 2014), and measured levels by qPCR (Fig 1G). In order to avoid overamplification during qPCR, we used an oligo‐dT‐containing primer with an AG anchor for limited cDNA production. Additionally, total RNA was reverse‐transcribed from *HNRNPR* ^+/+^ and −/− cells for quantification of tRNA. While tRNA levels were unchanged (Fig 1H), mRNA amounts were increased in *HNRNPR* ^−/−^ relative to +/+ cells (Fig 1I and J), in agreement with our FISH analysis. To investigate *de novo* synthesis of RNA, we metabolically labelled nascent RNA in *HNRNPR* ^+/+^ and −/− cells with 5‐ethynyl‐uridine (EU) followed by detection with click chemistry (Appendix Fig S4A and B). Compared to *HNRNPR* ^+/+^ cells, nuclear EU labelling was enhanced in −/− cells, indicating increased RNA synthesis. Thus, transcription is enhanced upon depletion of hnRNP R.

### Phosphorylation of RNA polymerase II is enhanced in hnRNP R knockout cells

**Figure 2:**
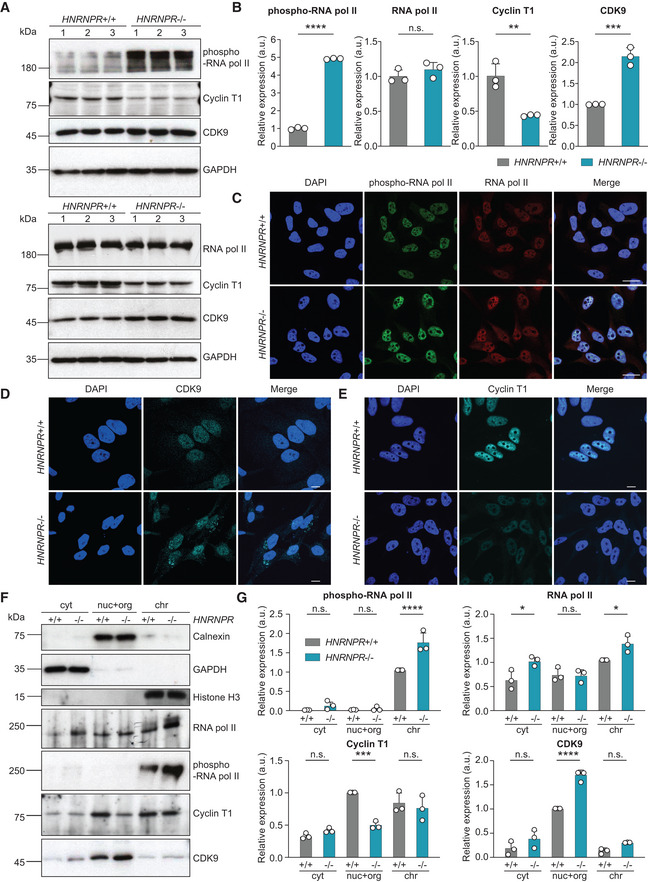
Increased Ser2 phosphorylation of RNA pol II in hnRNP R‐depleted cells AWestern blot analysis of Ser2‐phosphorylated and total RNA pol II, Cyclin T1, CDK9 and GAPDH protein levels.BQuantification of relative expression of Ser2‐phosphorylated and total RNA pol II, Cyclin T1 and CDK9 in (A). Data are mean with SD; **P ≤ 0.01, ***P ≤ 0.001, ****P ≤ 0.0001, n.s. not significant; unpaired two‐tailed *t*‐tests (*n* = 3 biological replicates).CImmunostaining with antibodies against Ser2‐phosphorylated and total RNA pol II. Scale bar: 20 μm.DImmunostaining with an antibody against CDK9. Scale bar: 10 μm.EImmunostaining with an antibody against Cyclin T1. Scale bar: 10 μm.FWestern blot analysis of subcellular fractions for Calnexin, GAPDH, histone H3, Ser2‐phosphorylated and total RNA pol II, Cyclin T1 and CDK9. Cyt, cytosol; nuc+org, nuclear soluble proteins and organelles; chr, chromatin‐associated proteins.GQuantification of relative expression of Ser2‐phosphorylated and total RNA pol II, Cyclin T1 and CDK9 in subcellular fractions in (F). Data are mean with SD; *P ≤ 0.05, ***P ≤ 0.001, ****P ≤ 0.0001, n.s. not significant; two‐way ANOVA with Sidak's multiple comparisons test (*n* = 3 biological replicates).

To obtain insights into the mechanism underlying increased transcription in hnRNP R‐deficient cells, we investigated activation of the transcriptional machinery. We observed increased Ser2 phosphorylation of RNA pol II in *HNRNPR* ^−/−^ cells, while total RNA pol II levels were unchanged (Fig 2A–C). This suggests that activation of RNA pol II through enhanced Ser2 phosphorylation facilitates the increased synthesis of mRNA seen in *HNRNPR* ^−/−^ cells. To validate the specificity of this finding, we overexpressed hnRNP R fused to EGFP at its C‐terminus in *HNRNPR* ^−/−^ cells. Compared to the expression of EGFP alone, expression of hnRNP R‐EGFP reduced Ser2 phosphorylation of RNA pol II in *HNRNPR* ^−/−^ cells back to normal levels (Appendix Fig S5A and B). Given that Ser2 is phosphorylated by P‐TEFb, we next investigated total levels of Cyclin T1 and CDK9. We observed that the amounts of Cyclin T1 were reduced, whereas those of CDK9 were increased by hnRNP R deficiency (Fig 2A, B, D and E). These changes in Cyclin T1 and CDK9 levels could be reversed upon expression of hnRNP R‐EGFP in *HNRNPR* ^−/−^ cells (Appendix Fig S5A and B).

To identify the subcellular region where these changes occur, we fractionated cells into a cytosolic fraction (cyt), a fraction containing nuclear soluble material and organelles (nuc+org) and a chromatin fraction (chr). As marker proteins, we used GAPDH for the cyt fraction, Calnexin for the nuc+org fraction and histone H3 for the chr fraction (Fig 2F). As expected, increased RNA pol II Ser2 phosphorylation in fractionated *HNRNPR* ^−/−^ cells was only detectable in the chr fraction (Fig 2F and G) where transcription takes place. In contrast, the decrease in Cyclin T1 levels and increase in CDK9 levels occurred in the nuc+org fraction of *HNRNPR* ^−/−^ cells (Fig 2F and G). This indicates that hnRNP R modulates transcription activation by regulating the levels of nuclear soluble CDK9. Stabilization and heterodimerization of CDK9 have previously been shown to involve the chaperones HSP70 and HSP90 (O'Keeffe *et al*, 2000). We observed that binding of CDK9 with HSP70 was unchanged and binding of CDK9 and HSP90 was reduced in *HNRNPR* ^−/−^ cells (Appendix Fig S6A–C). Interestingly, total amounts of HSP70 were increased in *HNRNPR* ^−/−^ cells, accompanying the increased levels of CDK9 (Appendix Fig S6D).

### hnRNP R regulates the association of P‐TEFb with 7SK

**Figure 3:**
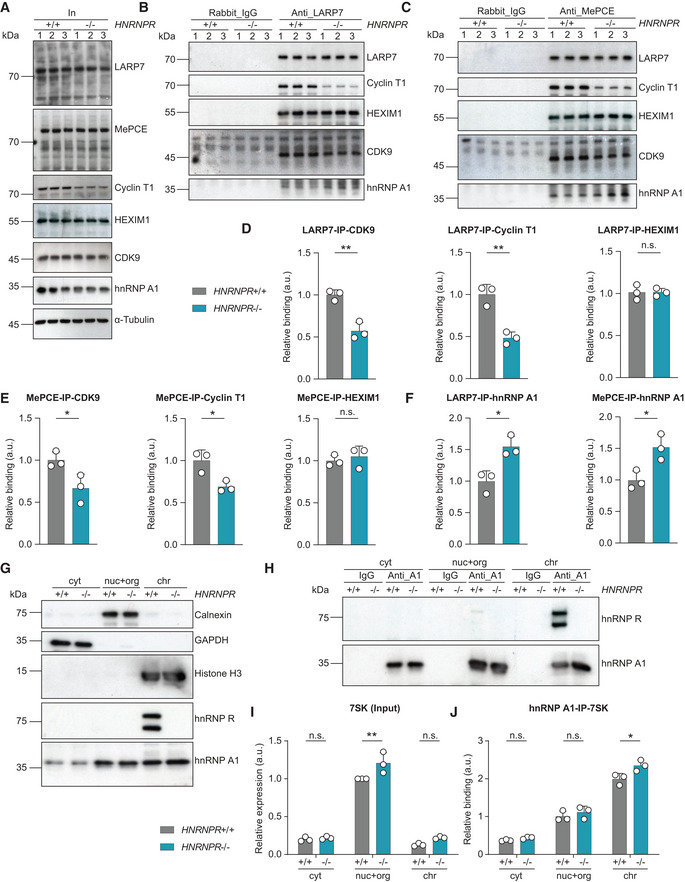
P‐TEFb is released from 7SK in hnRNP R‐deficient cells AWestern blot analysis of the indicated proteins in the input (In) lysates used for co‐immunoprecipitation.BWestern blot analysis of Cyclin T1, HEXIM1, CDK9 and hnRNP A1 co‐immunoprecipitated by an anti‐LARP7 antibody. Immunoprecipitation with rabbit‐IgG antibody was used as control.CWestern blot analysis of Cyclin T1, HEXIM1, CDK9, hnRNP A1 and LARP7 co‐immunoprecipitated by an anti‐MePCE antibody. Immunoprecipitation with rabbit‐IgG antibody was used as control.DQuantification of CDK9, Cyclin T1 and HEXIM1 co‐immunoprecipitating with LARP7 in (B). Data are mean with SD; **P ≤ 0.01, n.s. not significant; unpaired two‐tailed *t*‐tests (*n* = 3 biological replicates).EQuantification of CDK9, Cyclin T1 and HEXIM1 co‐immunoprecipitating with MePCE in (C). Data are mean with SD; *P ≤ 0.05, n.s. not significant; unpaired two‐tailed *t*‐tests (*n* = 3 biological replicates).FQuantification of hnRNP A1 co‐immunoprecipitating with LARP7 and MePCE in (B) and (C). Data are mean with SD; *P ≤ 0.05; unpaired two‐tailed *t*‐tests (*n* = 3 biological replicates).GWestern blot analysis of subcellular fractions for Calnexin, GAPDH, histone H3, hnRNP R and hnRNP A1. Cyt, cytosol; nuc+org, nuclear soluble proteins and organelles; chr, chromatin‐associated proteins.HWestern blot analysis of hnRNP R co‐immunoprecipitated by an anti‐hnRNP A1 antibody from subcellular fractions. Immunoprecipitation with mouse‐IgG antibody was used as control.IQuantification of 7SK RNA in subcellular fractions by qPCR. Data are mean with SD; **P ≤ 0.01, n.s. not significant; two‐way ANOVA with Sidak's multiple comparisons test (*n* = 3 biological replicates).JQuantification of 7SK RNA co‐immunoprecipitating with hnRNP A1 from subcellular fractions. Data are mean with SD; *P ≤ 0.05, n.s. not significant; two‐way ANOVA with Sidak's multiple comparisons test (*n* = 3 biological replicates). Source data are available online for this figure.

The P‐TEFb complex is inhibited by binding to 7SK, and hnRNP R might indirectly adjust their association by modifying the availability of 7SK. According to this model, deficiency of hnRNP R would be predicted to increase P‐TEFb sequestration by 7SK. To test this possibility, we immunopurified 7SK complexes from lysates of *HNRNPR* ^+/+^ and −/− cells using antibodies against MePCE or LARP7 and monitored co‐precipitation of CDK9, Cyclin T1 and HEXIM1 by immunoblotting (Fig 3A–C). Surprisingly, we observed that reduced amounts of CDK9 and Cyclin T1 were co‐purified with anti‐LARP7 or anti‐MePCE in *HNRNPR* ^−/−^ cells, whereas HEXIM1 co‐precipitation was unchanged (Fig 3D and E). This result indicates that P‐TEFb is released from 7SK upon hnRNP R depletion, contrasting the assumption that hnRNP deficiency leads to enhanced P‐TEFb interaction with 7SK.

To obtain insights into the mechanisms that might alter the association of P‐TEFb with 7SK in hnRNP R‐deficient cells, we first assessed whether hnRNP R associates with the P‐TEFb complex or HEXIM1, thereby stabilizing their binding to 7SK. However, while we observed an interaction between Cyclin T1, CDK9 and HEXIM1, we did not observe binding of hnRNP R to these components (Appendix Fig S7A–C). Next, we tested whether hnRNP R might be replaced by another RNA‐binding protein in *HNRNPR* ^−/−^ cells, thereby releasing P‐TEFb. For this purpose, we monitored the interaction of 7SK with hnRNP A1, an abundant nuclear hnRNP previously identified as 7SK interactor (Barrandon *et al*, 2007; Van Herreweghe *et al*, 2007; Ji *et al*, 2021). We observed enhanced co‐precipitation of hnRNP A1 with LARP7 and MePCE in *HNRNPR* ^−/−^ compared to +/+ cells (Fig 3F). This suggests that chronic depletion of hnRNP R leads to increased association of hnRNP A1 with 7SK, thereby dissociating P‐TEFb from 7SK. In agreement with this notion, hnRNP A1 was not associated with Cyclin T1 or CDK9 (Appendix Fig S7B and C).

To determine the subcellular site of 7SK interaction with hnRNP A1, we performed 7SK RNA immunoprecipitation with antibodies against hnRNP A1 from fractionated *HNRNPR* ^+/+^ and −/− cells (Fig 3G and H). While 7SK levels were highest in the nuc+org fraction as observed before (Ji *et al*, 2021), the association of 7SK with hnRNP A1 was increased in the chr fraction of *HNRNPR* ^−/−^ compared to +/+ cells (Fig 3I and J). We also observed that co‐precipitation of hnRNP R with hnRNP A1 in *HNRNPR* ^+/+^ cells was highest in the chr fraction (Fig 3H). This indicates that hnRNP A1 and hnRNP R are components of pre‐mRNPs, and that loss of hnRNP R leads to a re‐distribution of nuclear 7SK from the soluble fraction to chromatin‐associated hnRNP A1, thereby releasing P‐TEFb. Thus, P‐TEFb binding to 7SK is not *per se* determined by the availability of a single hnRNP but rather by the overall abundance of 7SK/hnRNP subcomplexes whose composition and localization is dynamically altered depending on individual hnRNP levels.

Finally, we investigated the release of P‐TEFb from 7SK by subjecting lysates of *HNRNPR* ^+/+^ and −/− cells to glycerol gradient ultracentrifugation and fractionation followed by immunoblotting for CDK9 (Appendix Fig S8). We observed a subtle shift of CDK9 towards the lighter fractions of the glycerol gradient, indicating increased amounts of free P‐TEFb. Nevertheless, the distribution of CDK9 in the heavier fractions containing large forms of P‐TEFb complexes appears unaffected. While this result is in agreement with our finding that the association of P‐TEFb with LARP7 and MePCE is mildly reduced in *HNRNPR* ^−/−^ compared to +/+ cells, it indicates the possibility that additional remodelling of CDK9 interactions with other proteins occurs upon hnRNP R depletion.

### BRD4 binding to P‐TEFb is modulated by hnRNP R

**Figure 4:**
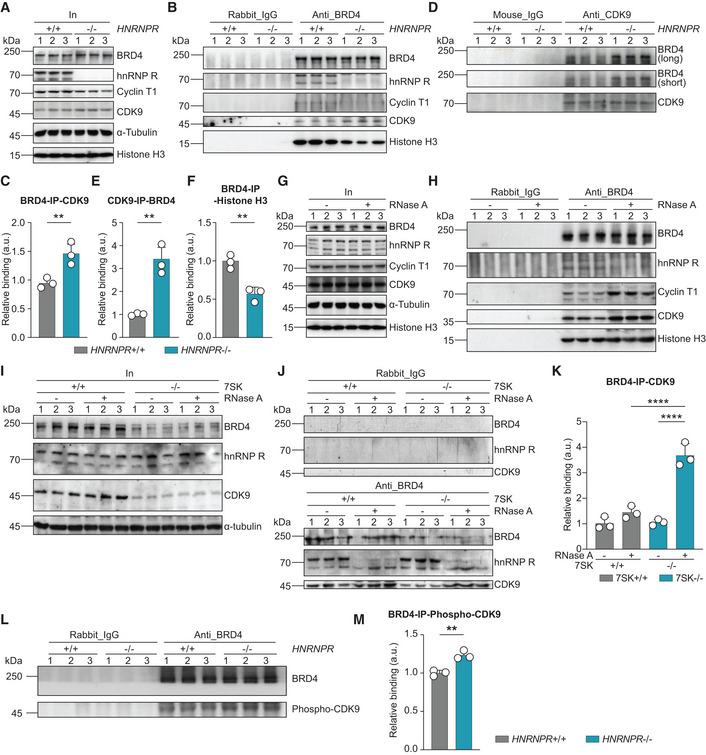
hnRNP R depletion enhances P‐TEFb binding to BRD4 AWestern blot analysis of BRD4, hnRNP R, Cyclin T1, CDK9, α‐Tubulin and histone H3 in the input lysates used for co‐immunoprecipitation.BWestern blot analysis of hnRNP R, Cyclin T1, CDK9 and histone H3 co‐immunoprecipitated by an anti‐BRD4 antibody. Immunoprecipitation with rabbit‐IgG antibody was used as control.CQuantification of CDK9 co‐immunoprecipitating with BRD4 in (B). Data are mean with SD; **P ≤ 0.01; unpaired two‐tailed *t*‐test (*n* = 3 biological replicates).DWestern blot analysis of BRD4 (long and short exposure) co‐immunoprecipitated by an anti‐CDK9 antibody. Immunoprecipitation with mouse‐IgG antibody was used as control.EQuantification of BRD4 co‐immunoprecipitating with CDK9 in (D). Data are mean with SD; **P ≤ 0.01; unpaired two‐tailed *t*‐test (*n* = 3 biological replicates).FQuantification of histone H3 co‐immunoprecipitating with BRD4 in (B). Data are mean with SD; **P ≤ 0.01; unpaired two‐tailed *t*‐test (*n* = 3 biological replicates).GWestern blot analysis of BRD4, hnRNP R, Cyclin T1, CDK9, α‐Tubulin and histone H3 in the input lysates used for co‐immunoprecipitation. Lysates were pre‐treated with RNase as indicated.HWestern blot analysis of hnRNP R, Cyclin T1, CDK9 and histone H3 co‐immunoprecipitated by an anti‐BRD4 antibody. Immunoprecipitation with rabbit‐IgG antibody was used as control.IWestern blot analysis of BRD4, hnRNP R, CDK9 and α‐Tubulin in the input lysates used for co‐immunoprecipitation.JWestern blot analysis of hnRNP R and CDK9 co‐immunoprecipitated by an anti‐BRD4 antibody. Immunoprecipitation with rabbit‐IgG antibody was used as control.KQuantification of CDK9 co‐immunoprecipitating with BRD4 in (J). Data are mean with SD; ****P ≤ 0.0001; two‐way ANOVA with Sidak's multiple comparisons test (*n* = 3 biological replicates).LWestern blot analysis of phospho‐CDK9(Thr186) co‐immunoprecipitated by an anti‐BRD4 antibody. Immunoprecipitation with rabbit‐IgG antibody was used as control.MQuantification of phospho‐CDK9(Thr186) co‐immunoprecipitating with BRD4 in (L). Data are mean with SD; **P ≤ 0.01; unpaired two‐tailed *t*‐test (*n* = 3 biological replicates).

The activity of P‐TEFb for transcriptional activation is mediated through interaction with BRD4, which binds to acetylated histones and thereby assists in the recruitment of P‐TEFb to sites of paused RNA pol II (Jang *et al*, 2005; Yang *et al*, 2005; Liu *et al*, 2013). By immunoprecipitation with an antibody against BRD4, we observed that binding of CDK9 to BRD4 was enhanced in *HNRNPR* ^−/−^ relative to +/+ cells (Fig 4A–C). Likewise, enhanced association between BRD4 and CDK9 was also detectable by anti‐CDK9 immunoprecipitation (Fig 4D and E). In contrast to CDK9, we did not observe increased binding of Cyclin T1 to BRD4 in hnRNP R‐deficient cells (Fig 4B), which, most likely, is a consequence of reduced Cyclin T1 expression (Fig 4A). Surprisingly, the association of BRD4 with histone H3 was reduced in *HNRNPR* ^−/−^ relative to +/+ cells (Fig 4F). This finding might be indicative for increased chromatin remodelling by BRD4, itself being a histone acetyltransferase, in hnRNP R‐deficient cells resulting in histone release from nucleosomes (Devaiah *et al*, 2016).

In agreement with a regulatory role of hnRNP R for BRD4, we found that BRD4 interacts with hnRNP R (Fig 4B, G and H). Following RNA removal by RNase treatment, the interaction of BRD4 with hnRNP R was reduced and, at the same time, CDK9 binding to BRD4 was enhanced (Fig 4G and H). We also observed increased association of Cyclin T1 with BRD4 upon RNA digestion (Fig 4G and H) congruent with previous observations that BRD4 interacts with both CDK9 and Cyclin T1 (Jang *et al*, 2005; Yang *et al*, 2005). As expected, BRD4 binding to histone H3 was independent of RNase treatment (Fig 4G and H).

The enhanced interaction between CDK9 and BRD4 upon RNA removal is likely a consequence of P‐TEFb release from 7SK by RNase treatment but, in addition, might involve reduced inhibition of BRD4 due to loss of its binding to hnRNP R. To distinguish between these possibilities, we generated 7SK knockout cells by inserting a transcriptional stop sequence for RNA pol III at the 5′ end of the 7SK gene (Appendix Fig S9A–F) and immunopurified BRD4 from lysates of 7SK^+/+^ and −/− cells treated with RNase (Fig 4I and J). Co‐purification of CDK9 with BRD4 from lysates of both 7SK^+/+^ as well as −/− cells was enhanced by RNA degradation (Fig 4K). This indicates that hnRNP R inhibits BRD4 binding to CDK9 in a 7SK‐independent manner. Compared to RNase‐treated 7SK^+/+^ cell lysate, BRD4 binding to CDK9 was even higher in treated 7SK^−/−^ lysate (Fig 4K). This finding might reflect the increased availability of hnRNP R for BRD4 inhibition in 7SK‐depleted cells. Finally, we investigated whether CDK9 bound to BRD4 in *HNRNPR* ^−/−^ cells is phosphorylated at Thr186, which is required for its activation (Baumli *et al*, 2008). We observed enhanced levels of phospho‐CDK9(Thr186) co‐immunoprecipitation with BRD4 in *HNRNPR* ^−/−^ cells (Fig 4L and M), indicating that it is in the active state. Together, these results indicate that hnRNP R negatively regulates transcription by inhibiting BRD4 binding to CDK9.

### Protein synthesis regulates transcription activation in an hnRNP R‐dependent manner

**Figure 5:**
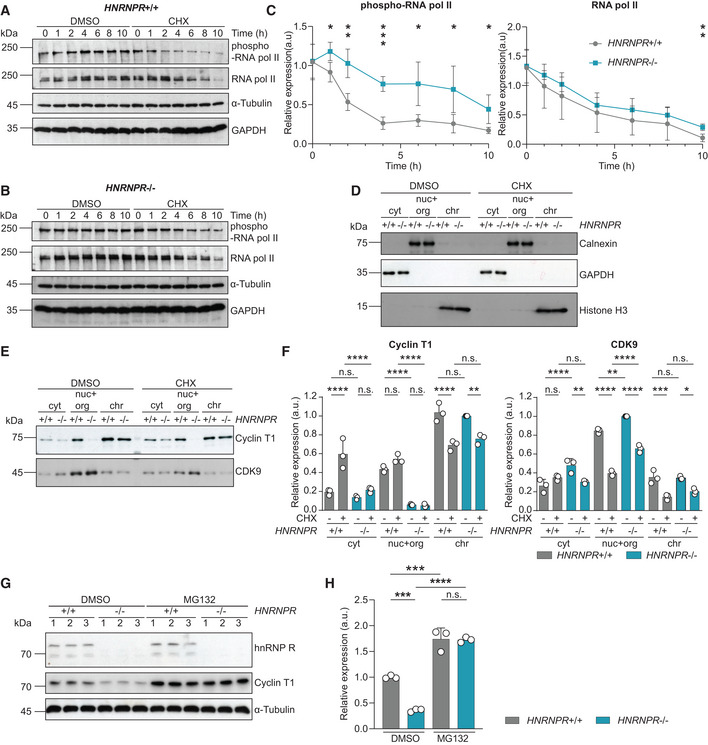
Translation inhibition regulates RNA pol II phosphorylation through hnRNP R‐dependent destabilization of CDK9 A, BWestern blot analysis of Ser2‐phosphorylated and total RNA pol II levels in *HNRNPR* ^+/+^ (A) and −/− (B) cells treated with DMSO or CHX for the indicated durations. GAPDH and α‐Tubulin served as loading control.CQuantification of relative expression levels of Ser2‐phosphorylated and total RNA pol II in (A, B). Data are mean with SD; *P ≤ 0.05, **P ≤ 0.01, ***P ≤ 0.001; unpaired two‐tailed *t*‐tests (*n* = 4 biological replicates).D, EWestern blot analysis of subcellular fractions from cells treated with DMSO or CHX for Calnexin, GAPDH and histone H3 (D), and Cyclin T1 and CDK9 (E). Cyt, cytosol; nuc+org, nuclear soluble proteins and organelles; chr, chromatin‐associated proteins.FQuantification of relative expression of Cyclin T1 and CDK9 in subcellular fractions in (E). Data are mean with SD; *P ≤ 0.05, **P ≤ 0.01, ***P ≤ 0.001, ****P ≤ 0.0001, n.s. not significant; one‐way ANOVA with Tukey's multiple comparisons test (*n* = 3 biological replicates).GWestern blot analysis of Cyclin T1 levels in *HNRNPR* ^+/+^ and −/− cells treated with DMSO or MG132. α‐Tubulin served as loading control.HQuantification of relative expression levels of Cyclin T1 in (G). Data are mean with SD; ***P ≤ 0.001, ****P ≤ 0.0001, n.s. not significant; two‐way ANOVA with Sidak's multiple comparisons test (*n* = 3 biological replicates).

Next, we investigated the consequences of translational inhibition on Ser2 phosphorylation of RNA pol II. For this purpose, we treated *HNRNPR* ^+/+^ and −/− cells with cycloheximide (CHX; 100 μg/ml) or DMSO for different durations ranging from 0 to 10 h and investigated the levels of total and Ser2‐phosphorylated RNA pol II by immunoblotting (Fig 5A and B). Total levels of RNA pol II continuously declined to similar extents in *HNRNPR* ^+/+^ and −/− cells with increasing duration of CHX treatment, indicating that the stability of RNA pol II is not altered by hnRNP R deficiency (Fig 5C). In contrast, while Ser2 phosphorylation of RNA pol II was rapidly lost following inhibition of protein synthesis in *HNRNPR* ^+/+^ cells, it was stabilized by the absence of hnRNP R (Fig 5C). This shows that the reduced transcriptional activation in response to translation inhibition is dependent on hnRNP R.

To investigate whether the enhanced fraction of Ser2‐phosphorylated RNA pol II in *HNRNPR* ^−/−^ cells might be a consequence of enhanced stability of the P‐TEFb complex components in the nucleus, we exposed *HNRNPR* ^+/+^ and −/− cells to CHX for 6 h and examined Cyclin T1 and CDK9 levels in cell fractions by immunoblotting (Fig 5D and E). While CDK9 was reduced in the nuc+org fraction following 6 h of CHX exposure, the remaining CDK9 levels in the nuc+org fraction were higher in *HNRNPR* ^−/−^ compared to +/+ cells (Fig 5F). This indicates that the stability of CDK9 is increased by hnRNP R deficiency. Thus, hnRNP R modulates the transcriptional response to changes in protein synthesis by destabilizing CDK9, thereby affecting the phosphorylation status of RNA pol II.

Cyclin T1, on the other hand, was already strongly reduced in the nuc+org fraction of *HNRNPR* ^−/−^ cells and not destabilized further by CHX treatment (Fig 5F). Cyclin T1 has previously been shown to be degraded by the proteasome (Budhiraja *et al*, 2013; Huang *et al*, 2021). This prompted us to investigate whether proteasomal degradation might contribute to the reduced Cyclin T1 levels we observed in *HNRNPR* ^−/−^ cells. To test this, we exposed *HNRNPR* ^+/+^ and −/− cells to the proteasome inhibitor MG132 and assessed total Cyclin T1 levels by immunoblotting (Fig 5G). We observed that Cyclin T1 levels were restored in *HNRNPR* ^−/−^ cells upon proteasome inhibition, indicating that Cyclin T1 is degraded following loss of hnRNP R (Fig 5H).

### Loss of hnRNP R promotes formation of CDK9/Cyclin K heterodimers

**Figure 6:**
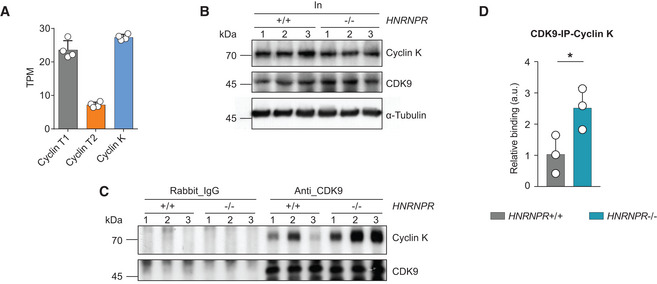
Enhanced association of CDK9 with Cyclin K in hnRNP R knockout cells ATPM expression values for mRNAs encoding Cylin T1, T2 and K from RNA‐seq data of wildtype HeLa cells (*n* = 4 biological replicates). Data are mean with SD (*n* = 4 biological replicates).BWestern blot analysis of Cyclin K, CDK9 and α‐Tubulin in the input lysates used for co‐immunoprecipitation.CWestern blot analysis of Cyclin K co‐immunoprecipitated by an anti‐CDK9 antibody. Immunoprecipitation with rabbit‐IgG antibody was used as control.DQuantification of Cyclin K co‐immunoprecipitating with CDK9 in (C). Data are mean with SD; *P ≤ 0.05; unpaired two‐tailed *t*‐test (*n* = 3 biological replicates).

Our results show that CDK9 levels are increased, whereas Cyclin T1 levels are reduced in *HNRNPR* ^−/−^ cells. Since CDK9 requires a Cyclin subunit for phosphorylation of RNA pol II, we asked whether binding of CDK9 to other Cyclins known to interact with CDK9 is altered in *HNRNPR* ^−/−^ cells. Among candidate interactors are Cyclin T2a/b and Cyclin K, which have been shown to form functional kinase complexes with CDK9 (Peng *et al*, 1998; Fu *et al*, 1999). To assess, which of these Cyclins is the most promising candidate we performed RNA‐seq on wild‐type HeLa cells (see below) and quantified the expression levels of the mRNAs encoding Cyclin T1, T2a/b and K as Transcripts Per Million (TPM). We found that the mRNAs encoding Cyclin T1 and Cyclin K have similar expression levels, while the mRNA encoding Cyclin T2a/b is present only at low levels (Fig 6A). For this reason we decided to investigate the interaction between CDK9 and Cyclin K in *HNRNPR* ^+/+^ and −/− cells by immunoprecipitation (Fig 6B and C). We observed that the association of Cyclin K with CDK9 was enhanced in *HNRNPR* ^−/−^ cells (Fig 6D), while total levels of Cyclin K were unchanged (Fig 6B). This finding suggests that the formation of an alternative CDK9 complex containing Cyclin K is enhanced in hnRNP R knockout cells.

### Transcriptome alterations are similar in hnRNP R‐ and 7SK‐deficient cells

**Figure 7:**
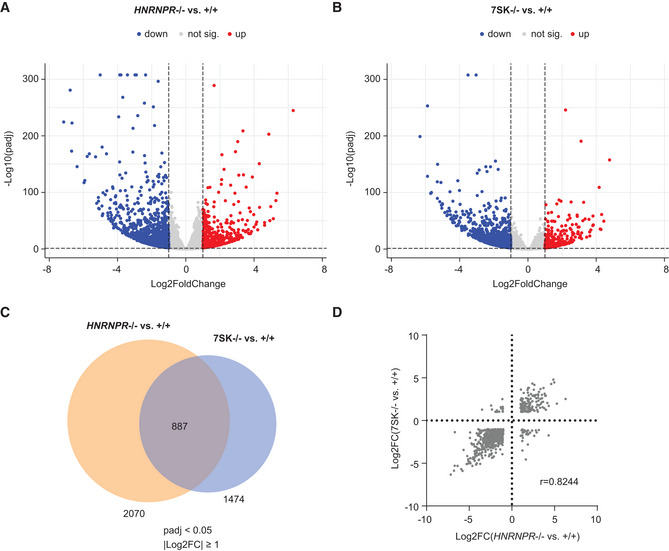
Transcriptome alterations in hnRNP R and 7SK knockout cells A, BVolcano plots showing the logarithmized adjusted *P*‐values (*P*_adj_) and fold changes of transcript alterations in *HNRNPR* ^−/−^ (A) and 7SK^−/−^ (B) cells relative to their +/+ controls (*n* = 4 biological replicates for *HNRNPR* ^+/+^, −/− and 7SK^+/+^; *n* = 2 biological replicates for 7SK^−/−^).CVenn diagram depicting numbers of transcripts significantly altered in *HNRNPR* ^−/−^ relative to +/+ cells, and in 7SK^−/−^ relative to +/+ cells, and their overlap.DScatter plot of the logarithmized fold changes (log2FC) of transcripts significantly altered in both *HNRNPR* ^−/−^ and 7SK^−/−^ cells relative to their +/+ controls.

Our results indicate that transcription in *HNRNPR* ^−/−^ is altered due to the release of P‐TEFb from 7SK. It can thus be hypothesized that transcripts changed upon hnRNP R loss might be altered in a similar manner in 7SK‐deficient cells, in which P‐TEFb is active due to the absence of 7SK. To test this possibility we performed RNA‐seq on *HNRNPR* ^+/+^ and −/−, and on 7SK^+/+^ and −/− cells (Dataset EV1). Principal component analysis (PCA) revealed that *HNRNPR* ^−/−^ and 7SK^−/−^ samples are located close to each other and distant to +/+ samples along PC1 representing 78% of variance (Appendix Fig S10). PC2 represents only 18% of variance and reflects transcriptomic differences between *HNRNPR* ^−/−^ and 7SK^−/−^ samples. To investigate this further, we quantified transcriptome alterations by differential expression analysis. We identified 2,070 significantly (*P*_adj_ < 0.05 and ¦log2FoldChange¦ ≥ 1) changed transcripts in *HNRNPR* ^−/−^ relative to +/+ cells, of which 1,408 transcripts were downregulated and 662 transcripts were upregulated (Fig 7A, Dataset EV2). In 7SK^−/−^ relative to +/+ cells, 1,474 significant transcript changes were detectable, of which 1,154 were downregulated and 320 were upregulated (Fig 7B, Dataset EV3). Among differentially expressed transcripts, 887 were altered in both *HNRNPR* ^−/−^ and 7SK^−/−^ cells relative to their +/+ controls (Fig 7C). Strikingly, for this subset, the direction and magnitude of transcript changes were highly correlated between the knockout cells (Fig 7D). This finding indicates that transcriptional alterations induced by P‐TEFb release from 7SK contribute to those caused by loss of hnRNP R.

## Discussion

7SK is an abundant RNA scaffold that regulates multiple nuclear soluble and chromatin‐associated protein complexes, most importantly the P‐TEFb complex. By doing so, 7SK exerts key functions for adjusting the transcriptional output globally. This function is not only important in proliferating cells responding to stress conditions, but also during the differentiation of precursor cells into postmitotic cells such as neurons (Cates *et al*, 2021; Studniarek *et al*, 2021). We demonstrate here that the 7SK interactor hnRNP R regulates P‐TEFb activity and thereby transcription in multiple ways: (i) through modulating the association of P‐TEFb with 7SK, (ii) by altering the availability of BRD4 for CDK9 binding, and (iii) by affecting the stability of CDK9. These mechanisms are most likely interconnected such that enhanced release of CDK9 from 7SK leads to increased binding to BRD4, and that stabilization of CDK9 further promotes the formation of CDK9 heterodimers with Cyclins to enable RNA pol II phosphorylation. We propose that, in combination, these mechanisms fine‐tune transcription globally in a concerted manner in response to altered transcriptional requirements that might occur in response to changing physiological demands or stress exposure.

We observed that CDK9 levels were increased in hnRNP R knockout cells, whereas Cyclin T1 levels were reduced due to proteasomal degradation. Since CDK9 requires a Cyclin to become activated, depletion of Cyclin T1 would be predicted to reduce functional P‐TEFb complexes. However, we found that, even though total levels of Cyclin T1 were reduced, the levels of Cyclin T1 in the chromatin fraction remained stable in *HNRNPR* ^−/−^ cells. Thus, we hypothesize that the increased amounts of CDK9 present in the nuclear soluble fraction of *HNRNPR* ^−/−^ cells can associate with BRD4, which then assembles fully functional P‐TEFb on chromatin. Indeed, a recent report has shown that P‐TEFb dissociates into monomers upon release from 7SK and requires BRD4 to re‐assemble (Zhou *et al*, 2022). Additionally, we observed that CDK9 association with Cyclin K is enhanced in hnRNP R‐deficient cells, indicating that increased levels of CDK9/Cyclin K complexes contribute to the enhanced RNA pol II phosphorylation in *HNRNPR* ^−/−^ cells. While our transcriptomics data indicate that Cyclin K is expressed at higher levels than Cyclin T2a/b, we cannot rule out the possibility that Cyclin T2a/b is stabilized through post‐translational mechanisms in *HNRNPR* ^−/−^ cells and thus might form additional complexes with CDK9 that can further phosphorylate RNA pol II. Either way, our results indicate that the loss of an RNA‐binding protein can induce a shift in CDK9 binding to different Cyclins, thereby adjusting its activity.

Importantly, we found that loss of hnRNP R leads to enhanced transcription, which contrasts the current model of 7SK functioning according to which P‐TEFb is inactivated by 7SK when a 7SK‐interacting hnRNP is depleted (Barrandon *et al*, 2007; Van Herreweghe *et al*, 2007). We observed that loss of hnRNP R leads to P‐TEFb release from 7SK, accompanied by increased hnRNP A1 binding to 7SK. Thus, hnRNP R normally prevents hnRNP A1 binding to 7SK, increasing 7SK availability for P‐TEFb sequestration. Considering that, apart from hnRNP R and A1, additional RNA‐binding proteins have been identified as 7SK interactors, our finding indicates that multiple 7SK/hnRNP subcomplexes co‐exist in a dynamic equilibrium, and that the balance between these subcomplexes ultimately determines the relative proportion of 7SK available for association with P‐TEFb. Notably, while the association of CDK9 and Cyclin T1 with LARP7 and MePCE was reduced in hnRNP R knockout cells, binding of HEXIM1 to LARP7 and MePCE was unaffected. This finding seemingly contrasts the notion that P‐TEFb binding to 7SK is mediated through HEXIM1 (Michels *et al*, 2004; Egloff *et al*, 2006). However, we observed that Cyclin T1 levels are reduced in *HNRNPR* ^−/−^ cells, and that CDK9 forms an alternative complex with Cyclin K. It is thus possible that, compared to CDK9/Cyclin T1, CDK9/Cyclin K complexes have a lower affinity to 7SK‐bound HEXIM1, thereby evading inhibition and allowing enhanced RNA pol II phosphorylation.

Heterogeneous nuclear ribonucleoproteins are often differentially expressed between tissues. For example, hnRNP R is enriched in the nervous system (Ghanawi *et al*, 2021). It is therefore possible that the relative amounts of individual 7SK/hnRNP complexes differ between distinct organs, contributing to cell‐type‐specific expression profiles. Additionally, the hnRNPs associated with 7SK have been implicated in neurodegenerative conditions such as amyotrophic lateral sclerosis and frontotemporal lobar degeneration (Kim *et al*, 2013; Deshaies *et al*, 2018; Gittings *et al*, 2019). Their dysfunction might affect 7SK binding and thereby dysbalance the association of 7SK with P‐TEFb and other proteins. This might not only lead to aberrant transcription but might also disturb other pathways such as axonal mRNA transport in neurons (Briese *et al*, 2018). We thus anticipate that the mechanism of P‐TEFb regulation described here might be of relevance to further elucidate the neuronal dysfunction underlying neurodegenerative disorders associated with defects in RNA processing.

## Materials and Methods

### HeLa cell culture

HeLa cells (Leibniz Institute DSMZ‐German Collection of Microorganisms and Cell Cultures GmbH, DSMZ no. ACC 57) were cultured at 37°C and 5% CO_2_ in high glucose Dulbecco's Modified Eagle Medium with GlutaMAX™ Supplement (DMEM; Gibco) supplemented with 1 mM sodium pyruvate (Gibco), 10% foetal calf serum (Linaris) and 1% Penicillin–Streptomycin (Gibco). Cells were passaged when they were 80–90% confluent. Cells tested negative for mycoplasma contamination.

### Generation of hnRNP R and 7SK knockout HeLa cell lines by prime editing

Knockout HeLa cells were generated by prime editing (Anzalone *et al*, 2019). The sgRNA sequence 5′‐ CAAGGTGCAAGAGTCCACAA‐3′ was identified in exon 4 of *HNRNPR* using the Broad Institute sgRNA design tool (https://portals.broadinstitute.org/gpp/public/analysis‐tools/sgrna‐design) and a pegRNA was designed for insertion of an in‐frame stop codon. For knockout of 7SK, the sgRNA sequence 5′‐GCTTGGGTACCTCGGATGTG‐3′ was used and a pegRNA was designed for insertion of a T(5) stretch. The pegRNAs were cloned into pU6‐pegRNA‐GG‐acceptor (Addgene Plasmid #132777; Anzalone *et al*, 2019) as following. One microgram pU6‐pegRNA‐GG‐acceptor was digested with Fast Digest Eco31I (IIs class; Thermo Fisher Scientific) in a 20 μl reaction. Following agarose gel electrophoresis, the vector backbone was gel‐purified using the NucleoSpin Gel and PCR Clean‐up kit (Macherey‐Nagel). Oligonucleotides pegRNA3‐1 and pegRNA3‐2 (Appendix Tables S1 and S2) were annealed and extended in a 30 μl PCR reaction containing 0.4 μM each of pegRNA3‐1 and pegRNA3‐2, 3 μl of 10× Extra Buffer, 0.33 mM of each dNTP and 1 U of Taq DNA polymerase (VWR) using 1 cycle of 94°C for 5 min 30 s, 60°C for 30 s and 72°C for 30 s. The pegRNA was assembled and inserted into pU6‐pegRNA‐GG‐acceptor in a 10 μl reaction containing 25 ng digested pU6‐pegRNA‐GG‐acceptor, 1.125 μl PCR reaction from the previous step, 45 nM pegRNA3‐3AGTGA (Appendix Table S1) or pegRNA3‐3TTTTT (Appendix Table S2) and 5 μl NEBuilder HiFi DNA Assembly Master Mix (NEB) and incubated at 37°C for 1 h.

For transfection, 10^5^ HeLa cells were seeded in a 12‐well plate in a volume of 0.5 ml of DMEM 24 h prior transfection. After 24 h, cells were co‐transfected with 750 ng of pCMV‐PE2 Plasmid (Addgene Plasmid #132775) and 250 ng of assembled pU6‐pegRNA‐GG‐acceptor using Lipofectamine 2000 (Invitrogen). Cells were harvested 72 h after transfection by trypsinization using TrypLE™ Express Enzyme (Gibco), counted and diluted to nine cells per ml DMEM medium. One hundred microlitres of cell suspension was added per well of a 96‐well plate. Cells were grown for 7–10 days to allow formation of colonies from single cells. Colonies were trypsinized, transferred to a 48‐well plate and grown for another 7 days for cell expansion. Then, cells in each well were trypsinized and split into two wells of a 24‐well plate. One of these wells was used for genotyping with primers listed in Appendix Tables S1 and S2.

### Proliferation

For the wound healing assay, 2 × 10^5^ HeLa cells were grown on coverslips for 2 days. A scratch was inflicted with a pipette tip in the middle of the coverslip and images were taken at different time points afterwards. The area not covered by cells was quantified with ImageJ as part of the Fiji package (Schindelin *et al*, 2012). For quantification of cell numbers, 10^5^ HeLa cells were seeded in T175 flasks. Cells were trypsinized on different days and counted with a Neubauer chamber.

### mRNA quantification

Levels of mRNA were quantified by a Smart‐seq2‐based approach as described before (Picelli *et al*, 2014; Jonasson *et al*, 2020). Briefly, RNA was purified from 10 × 10^6^ cultured cells using the NucleoSpin RNA kit (Macherey‐Nagel) and eluted in 60 μl water. Total RNA was reverse‐transcribed in a 20 μl reaction containing 10 μl RNA, 5 μM random hexamer, 4 μl 5× Reaction Buffer, 20 U RiboLock RNase Inhibitor, 1 mM each dNTP and 400 U RevertAid M‐MuLV RT enzyme (Thermo Fisher Scientific) at 25°C for 5 min, 37°C for 1 h and 70°C for 5 min. Two microlitres of the first strand reverse transcription reaction was then used for quantification of tRNA levels by qPCR. For reverse transcription of mRNA, 10.3 μl total RNA was first pre‐incubated with 10 pmol oligo‐dT30AG and 1 μl of 10 mM dNTP mix in a total volume of 12.3 μl at 72°C for 3 min and then placed on ice. Then, 7.7 μl reverse transcription mix was added containing 10 U RiboLock RNase inhibitor, 4 μl 5× First‐Strand Buffer, 0.5 μl of 100 mM DTT, 2 μl of 5 M betaine, 0.06 μl of 1 M MgCl_2_, 10 pmol TSO and 100 U SuperScript II RT enzyme (Invitrogen) and incubated at 42°C for 90 min, 10 cycles of 50°C for 2 min, 42°C for 2 min, and 70°C for 15 min. Two microlitres undiluted first‐strand reaction, or 2 μl of first‐strand reaction diluted 1:1, 1:20 or 1:200 in water were used for qPCR with ISPCR primer. Relative mRNA levels were calculated by normalization to tRNA using the 2^−ΔΔCt^ method. Oligonucleotide sequences are listed in Appendix Table S3.

### hnRNP R overexpression

An expression plasmid for hnRNP R was generated as following. Total RNA was extracted from HEK293TN cells with the NucleoSpin RNA kit (Macherey‐Nagel) and 1 μg RNA was reverse‐transcribed with oligo‐dT18 using the First Strand cDNA Synthesis Kit (Thermo Fisher Scientific). The coding sequence of full‐length hnRNP R was then PCR‐amplified from the cDNA using the KAPA HiFi HotStart ReadyMix (Roche) and sub‐cloned into pJET1.2 using the CloneJET PCR Cloning Kit (Thermo Fisher Scientific). Using primers listed in Appendix Table S4, the hnRNP R coding sequence with overhangs was amplified from pJET1.2‐hnRNP R, and the EGFP coding sequence with overhangs was amplified from a pcDNA3‐EGFP expression plasmid. PCR products were subjected to DpnI (Thermo Fisher Scientific) digestion and, using the NEBuilder HiFi DNA Assembly Master Mix (NEB), were inserted into pcDNA3 digested with BamHI (Thermo Fisher Scientific) to generate pcDNA3‐hnRNP R‐EGFP. For transfection, plasmids were produced using the NucleoBond Xtra Midi kit (Macherey‐Nagel) and transfected using Lipofectamine 2000 (Thermo Fisher Scientific) as following. 2.5 × 10^6^ HeLa cells were seeded in a T75 flask and after 24 h transfected with 20 μg pcDNA3‐hnRNP R‐EGFP or 15.26 μg pcDNA3‐EGFP. Total protein was extracted 48 h after transfection for Western blot analysis using antibodies listed in Appendix Table S5.

### Immunofluorescence staining

For immunofluorescence staining, HeLa cells were grown on 10 mm glass coverslips for 2 days. Cells were washed three times with pre‐warmed phosphate‐buffered saline (PBS), fixed with 4% paraformaldehyde (PFA; Thermo Fisher Scientific) for 20 min at room temperature followed by three washes with pre‐warmed PBS. For permeabilization, 0.3% Triton X‐100 was applied for 20 min at room temperature followed by three washes with pre‐warmed PBS. For reduction of unspecific binding, cells were treated with 10% horse serum and 2% bovine serum albumin (BSA) in Tris‐buffered saline with Tween 20 (TBS‐T) for 0.5 h at room temperature. The primary antibody was applied overnight at 4°C followed by three washes with TBS‐T and incubation with fluorescently labelled secondary antibodies in TBS‐T for 1 h at room temperature. Cells were washed three times with TBS‐T at room temperature, incubated with DAPI (Sigma‐Aldrich) diluted 1:1,000 in PBS for 10 min and washed once with water. Cells were embedded with Aqua‐Poly/Mount (Polysciences). The primary and secondary antibodies used for immunostaining are listed in Appendix Table S5.

### Poly(A) FISH

HeLa cells were grown on 10 mm coverslips for 2 days, washed once with PBS and fixed with 4% PFA for 10 min. Following removal of PFA, cells were incubated with 100% cold methanol for 10 min and then with 70% ethanol for 10 min. Ethanol was aspirated and cells were incubated with 1 M Tris pH 8.0 (Thermo Scientific) for 5 min. While cells were incubated in Tris, oligo‐dT probe [cy3‐T(+T) TT(+T) TT(+T) TT(+T) TT(+T) TT(+T) TT(+T), where (+T) is LNA] was diluted to a final concentration of 2 ng/μl in hybridization buffer [2× SSC (Thermo Fisher Scientific), 0.1 mg/ml yeast tRNA (Sigma‐Aldrich), 0.005% Ultrapure™ BSA (50 mg/ml) (Thermo Scientific), 10% dextran sulphate (Sigma‐Aldrich) and 25% formamide (Sigma‐Aldrich)]. Tris was aspirated and cells were incubated with diluted probe at 37°C for 5 h. After hybridization, cells were washed once with 4× SSC, once with 2× SSC and incubated with DAPI (Sigma‐Aldrich) diluted 1:1,000 in Dulbecco's phosphate‐buffered saline (DPBS; Sigma‐Aldrich) for 10 min. Following DAPI staining, cells were washed twice with water and coverslips were mounted.

### 5‐ethynyl‐uridine labelling

HeLa cells grown on 10 mm coverslips for 2 days were treated with 1 mM EU (Jena Bioscience) for 6 h. As control, cells were co‐treated with 2 μM actinomycin D. Cells were washed once with PBS, fixed with 4% PFA for 15 min and washed once with 3% BSA in PBS. Cells were permeabilized with 0.5% Triton X‐100 in PBS for 15 min and washed once with 3% BSA in PBS. EU was detected at room temperature using the Click‐iT Cell Reaction Buffer Kit (Thermo Fisher Scientific) with Cy3‐Azide (Jena Bioscience) at 3.33 μM final concentration. Cells were washed with 3% BSA in PBS, stained with DAPI (1:1,000 in PBS), washed once with water and mounted.

### Confocal microscopy

Images were acquired on an Olympus Fluoview 1000 confocal system equipped with four excitation lasers: a 405 nm, a 473 nm, a 559 nm and a 635 nm laser. A 60× objective (oil differential interference contrast, numerical aperture: 1.35) was used for image acquisition. For quantification of immunofluorescence signals, raw images were projected using ImageJ/Fiji as average intensity and mean grey values were measured after background subtraction.

### Total protein extraction

HeLa cells were washed once with ice‐cold DPBS (without MgCl_2_, CaCl_2_; Sigma‐Aldrich) and collected by scraping. Cells were lysed in 1 ml lysis RIPA buffer (20 mM Tris–HCl pH 7.5, 150 mM NaCl, 1 mM Na_2_EDTA, 1 mM EGTA, 1% NP‐40, 1% sodium deoxycholate, 2.5 mM sodium pyrophosphate, 1 mM β‐glycerophosphate) on ice for 15 min. Lysates were centrifuged at 16,000 *g* for 15 min at 4°C and the supernatant was transferred into a new tube. Protein concentration was measured by Pierce BCA Protein Assay Kit (Thermo Fisher Scientific). Lysates were dissolved in 5× Laemmli buffer (300 mM Tris–HCl pH 6.8, 10% SDS, 50% glycerol, 0.05% Bromophenol Blue, 100 mM DTT) and analysed by Western blotting using antibodies listed in Appendix Table S5.

### Protein stability assay

For protein stability assays, 2 × 10^6^ cells were grown in 10 cm dishes for 2 days. Then, the medium was replaced with fresh medium containing 100 μg/ml cycloheximide or DMSO as control and cells were incubated for the indicated durations prior to harvesting. Total protein was extracted for Western blot analysis using antibodies listed in Appendix Table S5.

### Proteasomal inhibition

2.5 × 10^6^ cells were grown in T75 flasks for 2 days. Cells were exposed to DMSO as control or MG132 at a final concentration of 10 μM for 4 h. Total protein was extracted for Western blot analysis using antibodies listed in Appendix Table S5.

### Subcellular fractionation

Cell fractionation was performed as described previously (Ji *et al*, 2021). Briefly, 2 × 10^6^ HeLa cells were grown in 10 cm dishes for 2 days. Cells were washed once with ice‐cold DPBS and cytosolic proteins were extracted by adding 4 ml lysis buffer A (10 mM HEPES pH 7.0, 100 mM KCl, 5 mM MgCl_2_) containing 150 μg/ml digitonin and incubation for 10 min at 4°C. The lysate was then centrifuged at 2,000 *g* for 5 min at 4°C. The supernatant contains the cytosolic fraction (cyt). The cells remaining on the dish were washed once with ice‐cold DPBS and lysed in 4 ml lysis buffer B (10 mM HEPES pH 7.0, 100 mM KCl, 5 mM MgCl_2_, 0.5% NP‐40) for 15 min on ice. The lysate was centrifuged at 20,000 *g* for 15 min at 4°C and the supernatant containing nuclear soluble and organellar proteins (nuc+org) was transferred into a new tube. The pellet was washed once with ice‐cold DPBS, dissolved in 4 ml lysis buffer B and sonicated. This chromatin fraction (chr) contains nuclear insoluble proteins. The same volume of each fraction was used for Western blotting or RNA extraction for qPCR. For co‐immunoprecipitation, 800 μl of each fraction was used. The antibodies used for Western blotting are listed in Appendix Table S5.

### Immunoprecipitation

Cells were washed once with ice‐cold DPBS, collected by scraping and lysed in 1 ml lysis buffer (10 mM HEPES pH 7.0, 100 mM KCl, 5 mM MgCl_2_, 0.5% NP‐40) on ice for 15 min. Lysates were centrifuged at 20,000 *g* for 15 min at 4°C. For co‐immunoprecipitation of histone H3, lysates were sonicated prior to centrifugation. Ten microlitres of Dynabeads Protein G or A (depending on the antibody species; Thermo Fisher Scientific) and 1 μg antibody or IgG control were added to 200 μl lysis buffer and rotated for 30–40 min at room temperature. Then 200 μl lysate was added to the antibody‐bound beads and rotated for 2 h at 4°C. For RNase treatment, 3 μl RNase A (Thermo Fisher Scientific) was added to 200 μl lysate and incubated at 37°C for 10 min before proceeding with co‐immunoprecipitation. Beads were washed twice with lysis buffer and proteins were eluted in 1× Laemmli buffer. Proteins were size‐separated by SDS‐PAGE and analysed by Western blotting. The intensity of protein bands was measured with ImageJ/Fiji, and co‐immunoprecipitation was quantified by normalization to input and to the efficiency of bait protein purification. The antibodies used for immunoprecipitation and Western blotting are listed in Appendix Table S5.

### Glycerol gradient ultracentrifugation

HeLa cells were lysed in lysis buffer (10 mM HEPES pH 7.0, 100 mM KCl, 5 mM MgCl_2_, 0.5% NP‐40) and protein concentration was measured by Pierce BCA Protein Assay Kit (Thermo Fisher Scientific). Lysates were diluted to a final concentration of 1.5 μg/μl and 200 μl were layered onto a 5–45% glycerol gradient (10 mM HEPES pH 7.0, 100 mM KCl, 5 mM MgCl_2_). The gradients were run in a SW 60 Ti rotor at 45,000 rpm for 16 h. The gradient was fractionated and the same volume of each fraction was used for Western blotting using antibodies listed in Appendix Table S5.

### RNA‐seq

RNA was extracted from four biological replicates each of *HNRNPR* ^+/+^ and −/− cells, from four biological replicates of 7SK^+/+^ cells and from three biological replicates of 7SK^−/−^ cells using the NucleoSpin RNA kit (Macherey‐Nagel). RNA quality was checked using a 2100 Bioanalyzer with the RNA 6000 Nano kit (Agilent Technologies). The RIN for all samples was > 9.6. cDNA libraries were prepared from 300 ng of total RNA with TruSeq mRNA Stranded Library Prep Kit from Illumina according to manufacturer's instructions (1/2 volume). For 7SK^−/−^ cells, replicate 2 RNA was sequenced twice as technical replicates. Libraries were quantified by QubitTM Flex Fluorometer (Thermo Fisher Scientific) and quality was checked using Fragment Analyzer system 5200 with DNF‐474 HS NGS Kit (Agilent). Sequencing of pooled libraries, spiked with 1% PhiX control library, was performed at 27–33 million reads/sample in single‐end mode with 75 nt read length on the NextSeq 500 platform (Illumina). Demultiplexed FASTQ files were generated with bcl2fastq2 v2.20.0.422 (Illumina).

To assure high sequence quality, Illumina reads were quality‐ and adapter‐trimmed via Cutadapt (Martin, 2011) version 2.5 using a cut‐off Phred score of 20 in NextSeq mode, and reads without any remaining bases were discarded (command line parameters: –nextseq‐trim = 20 ‐m 1 ‐a AGATCGGAAGAGCACACGTCTGAACTCCAGTCAC). Processed reads were subsequently mapped to the human genome (GRCh38.p13 primary assembly and mitochondrion) using STAR v2.7.2b with default parameters based on RefSeq annotation version 109.20210226 for GRCh38.p13 (Dobin *et al*, 2013). Read counts on exon level summarized for each gene were generated using featureCounts v1.6.4 from the Subread package (Liao *et al*, 2014). Multi‐mapping and multi‐overlapping reads were counted strand‐specific and reversely stranded with a fractional count for each alignment and overlapping feature (command line parameters: ‐s 2 ‐t exon ‐M ‐O ‐‐fraction). The count output was utilized to identify differentially expressed genes using DESeq2 (Love *et al*, 2014) version 1.24.0. For 7SK^−/−^ cells, replicate 1 was identified as outlier and therefore removed from the analysis. Read counts were normalized by DESeq2 and fold‐change shrinkage was applied by setting the parameter ‘betaPrior = TRUE’. PCA was conducted using regularized log (rlog)‐transformed counts with the plotPCA function from DESeq2 and visualized via ggplot2 version 3.3.2 (Appendix Fig S10). Read counts from 7SK^−/−^ technical replicates 2 and 3 (RN7SK_KO2 and RN7SK_KO3) were collapsed to a column RN7SK_KO2_3 using function collapseReplicates on the DESeqDataSet. Differential expression of genes was assumed at an adjusted *P*‐value (*P*_adj_) after Benjamini–Hochberg correction < 0.05 and ¦log2FoldChange¦ ≥ 1. Volcano plots for visualization of DESeq2 results were generated using the EnhancedVolcano Bioconductor R package (Blighe *et al*, 2018) version 1.2.0.

### qPCR

Unless stated otherwise, RNA was extracted from HeLa cells using TRIzol (Thermo Fisher Scientific) and reverse‐transcribed with random hexamers using the First Strand cDNA Synthesis Kit (Thermo Fisher Scientific). Reverse transcription reactions were diluted 1:5 in water and qPCR reactions were set up with Luminaris HiGreen qPCR Master Mix (Thermo Fisher Scientific) on a LightCycler® 96 (Roche). Relative expression was calculated using the ΔΔCt method. Primers are listed in Appendix Table S6.

## Author contributions

**Changhe Ji:** Conceptualization; formal analysis; investigation; visualization. **Chunchu Deng:** Investigation. **Katharina Antor:** Investigation. **Thorsten Bischler:** Software; visualization. **Cornelius Schneider:** Investigation. **Utz Fischer:** Resources; supervision; funding acquisition. **Michael Sendtner:** Conceptualization; resources; supervision; funding acquisition; methodology; writing – original draft; project administration; writing – review and editing. **Michael Briese:** Conceptualization; formal analysis; supervision; funding acquisition; visualization; methodology; writing – original draft; project administration; writing – review and editing.

In addition to the CRediT author contributions listed above, the contributions in detail are:

CJ generated the knockout cell lines and performed the experiments. CD performed the confocal microscopy analysis. KA generated the hnRNP R overexpression construct. TB conducted the bioinformatics analysis of the RNA‐seq data. CS performed the glycerol gradient ultracentrifugation under supervision of UF. MS and MB supervised the study. MB prepared the manuscript with contributions from all authors.

## Disclosure and competing interests statement

The authors declare that they have no conflict of interest.

## Supporting information



Appendix

Dataset EV1

Dataset EV2

Dataset EV3

Source Data for Appendix

Source Data for Figure 3

## Data Availability

The RNA‐seq data are accessible in NCBI's Gene Expression Omnibus (Edgar *et al*, 2002) through GEO Series accession number GSE202720 (https://www.ncbi.nlm.nih.gov/geo/query/acc.cgi?acc=GSE202720).

## References

[embr202255432-bib-0001] Anzalone AV , Randolph PB , Davis JR , Sousa AA , Koblan LW , Levy JM , Chen PJ , Wilson C , Newby GA , Raguram A *et al* (2019) Search‐and‐replace genome editing without double‐strand breaks or donor DNA. Nature 576: 149–157 31634902 10.1038/s41586-019-1711-4 PMC6907074

[embr202255432-bib-0002] Barrandon C , Bonnet F , Nguyen VT , Labas V , Bensaude O (2007) The transcription‐dependent dissociation of P‐TEFb‐HEXIM1‐7SK RNA relies upon formation of hnRNP‐7SK RNA complexes. Mol Cell Biol 27: 6996–7006 17709395 10.1128/MCB.00975-07 PMC2168891

[embr202255432-bib-0003] Baumli S , Lolli G , Lowe ED , Troiani S , Rusconi L , Bullock AN , Debreczeni JE , Knapp S , Johnson LN (2008) The structure of P‐TEFb (CDK9/cyclin T1), its complex with flavopiridol and regulation by phosphorylation. EMBO J 27: 1907–1918 18566585 10.1038/emboj.2008.121 PMC2486423

[embr202255432-bib-0004] Blighe K , Rana S , Lewis M (2018) EnhancedVolcano: publication‐ready volcano plots with enhanced colouring and labeling. https://github.com/kevinblighe/EnhancedVolcano

[embr202255432-bib-0005] Briese M , Saal‐Bauernschubert L , Ji C , Moradi M , Ghanawi H , Uhl M , Appenzeller S , Backofen R , Sendtner M (2018) hnRNP R and its main interactor, the noncoding RNA 7SK, coregulate the axonal transcriptome of motoneurons. Proc Natl Acad Sci USA 115: E2859–E2868 29507242 10.1073/pnas.1721670115 PMC5866599

[embr202255432-bib-0006] Briese M , Sendtner M (2021) Keeping the balance: the noncoding RNA 7SK as a master regulator for neuron development and function. Bioessays 43: e2100092 34050960 10.1002/bies.202100092

[embr202255432-bib-0007] Budhiraja S , Famiglietti M , Bosque A , Planelles V , Rice AP (2013) Cyclin T1 and CDK9 T‐loop phosphorylation are downregulated during establishment of HIV‐1 latency in primary resting memory CD4^+^ T cells. J Virol 87: 1211–1220 23152527 10.1128/JVI.02413-12 PMC3554045

[embr202255432-bib-0008] Cates K , McCoy MJ , Kwon JS , Liu Y , Abernathy DG , Zhang B , Liu S , Gontarz P , Kim WK , Chen S *et al* (2021) Deconstructing stepwise fate conversion of human fibroblasts to neurons by microRNAs. Cell Stem Cell 28: 127–140 32961143 10.1016/j.stem.2020.08.015 PMC7796891

[embr202255432-bib-0009] Deshaies JE , Shkreta L , Moszczynski AJ , Sidibe H , Semmler S , Fouillen A , Bennett ER , Bekenstein U , Destroismaisons L , Toutant J *et al* (2018) TDP‐43 regulates the alternative splicing of hnRNP A1 to yield an aggregation‐prone variant in amyotrophic lateral sclerosis. Brain 141: 1320–1333 29562314 10.1093/brain/awy062 PMC5917749

[embr202255432-bib-0010] Devaiah BN , Case‐Borden C , Gegonne A , Hsu CH , Chen Q , Meerzaman D , Dey A , Ozato K , Singer DS (2016) BRD4 is a histone acetyltransferase that evicts nucleosomes from chromatin. Nat Struct Mol Biol 23: 540–548 27159561 10.1038/nsmb.3228 PMC4899182

[embr202255432-bib-0011] Dobin A , Davis CA , Schlesinger F , Drenkow J , Zaleski C , Jha S , Batut P , Chaisson M , Gingeras TR (2013) STAR: ultrafast universal RNA‐seq aligner. Bioinformatics 29: 15–21 23104886 10.1093/bioinformatics/bts635 PMC3530905

[embr202255432-bib-0012] Edgar R , Domrachev M , Lash AE (2002) Gene expression omnibus: NCBI gene expression and hybridization array data repository. Nucleic Acids Res 30: 207–210 11752295 10.1093/nar/30.1.207 PMC99122

[embr202255432-bib-0013] Egloff S , Van HE , Kiss T (2006) Regulation of polymerase II transcription by 7SK snRNA: two distinct RNA elements direct P‐TEFb and HEXIM1 binding. Mol Cell Biol 26: 630–642 16382153 10.1128/MCB.26.2.630-642.2006 PMC1346915

[embr202255432-bib-0014] Fu TJ , Peng J , Lee G , Price DH , Flores O (1999) Cyclin K functions as a CDK9 regulatory subunit and participates in RNA polymerase II transcription. J Biol Chem 274: 34527–34530 10574912 10.1074/jbc.274.49.34527

[embr202255432-bib-0015] Ghanawi H , Hennlein L , Zare A , Bader J , Salehi S , Hornburg D , Ji C , Sivadasan R , Drepper C , Meissner F *et al* (2021) Loss of full‐length hnRNP R isoform impairs DNA damage response in motoneurons by inhibiting Yb1 recruitment to chromatin. Nucleic Acids Res 49: 12284–12305 34850154 10.1093/nar/gkab1120 PMC8643683

[embr202255432-bib-0016] Gittings LM , Foti SC , Benson BC , Gami‐Patel P , Isaacs AM , Lashley T (2019) Heterogeneous nuclear ribonucleoproteins R and Q accumulate in pathological inclusions in FTLD‐FUS. Acta Neuropathol Commun 7: 18 30755280 10.1186/s40478-019-0673-y PMC6371513

[embr202255432-bib-0017] Huang F , Nguyen TT , Echeverria I , Rakesh R , Cary DC , Paculova H , Sali A , Weiss A , Peterlin BM , Fujinaga K (2021) Reversible phosphorylation of cyclin T1 promotes assembly and stability of P‐TEFb. eLife 10: e68473 34821217 10.7554/eLife.68473 PMC8648303

[embr202255432-bib-0018] Jang MK , Mochizuki K , Zhou M , Jeong HS , Brady JN , Ozato K (2005) The bromodomain protein Brd4 is a positive regulatory component of P‐TEFb and stimulates RNA polymerase II‐dependent transcription. Mol Cell 19: 523–534 16109376 10.1016/j.molcel.2005.06.027

[embr202255432-bib-0019] Ji C , Bader J , Ramanathan P , Hennlein L , Meissner F , Jablonka S , Mann M , Fischer U , Sendtner M , Briese M (2021) Interaction of 7SK with the Smn complex modulates snRNP production. Nat Commun 12: 1278 33627647 10.1038/s41467-021-21529-1 PMC7904863

[embr202255432-bib-0020] Jonasson E , Andersson L , Dolatabadi S , Ghannoum S , Aman P , Stahlberg A (2020) Total mRNA quantification in single cells: sarcoma cell heterogeneity. Cells 9: 759 10.3390/cells9030759 PMC7140709 32204559

[embr202255432-bib-0021] Kim HJ , Kim NC , Wang YD , Scarborough EA , Moore J , Diaz Z , MacLea KS , Freibaum B , Li S , Molliex A *et al* (2013) Mutations in prion‐like domains in hnRNPA2B1 and hnRNPA1 cause multisystem proteinopathy and ALS. Nature 495: 467–473 23455423 10.1038/nature11922 PMC3756911

[embr202255432-bib-0022] Krueger BJ , Jeronimo C , Roy BB , Bouchard A , Barrandon C , Byers SA , Searcey CE , Cooper JJ , Bensaude O , Cohen EA *et al* (2008) LARP7 is a stable component of the 7SK snRNP while P‐TEFb, HEXIM1 and hnRNP A1 are reversibly associated. Nucleic Acids Res 36: 2219–2229 18281698 10.1093/nar/gkn061 PMC2367717

[embr202255432-bib-0023] Liao Y , Smyth GK , Shi W (2014) featureCounts: an efficient general purpose program for assigning sequence reads to genomic features. Bioinformatics 30: 923–930 24227677 10.1093/bioinformatics/btt656

[embr202255432-bib-0024] Liu W , Ma Q , Wong K , Li W , Ohgi K , Zhang J , Aggarwal A , Rosenfeld MG (2013) Brd4 and JMJD6‐associated anti‐pause enhancers in regulation of transcriptional pause release. Cell 155: 1581–1595 24360279 10.1016/j.cell.2013.10.056 PMC3886918

[embr202255432-bib-0025] Love MI , Huber W , Anders S (2014) Moderated estimation of fold change and dispersion for RNA‐seq data with DESeq2. Genome Biol 15: 550 25516281 10.1186/s13059-014-0550-8 PMC4302049

[embr202255432-bib-0026] Markert A , Grimm M , Martinez J , Wiesner J , Meyerhans A , Meyuhas O , Sickmann A , Fischer U (2008) The La‐related protein LARP7 is a component of the 7SK ribonucleoprotein and affects transcription of cellular and viral polymerase II genes. EMBO Rep 9: 569–575 18483487 10.1038/embor.2008.72 PMC2427381

[embr202255432-bib-0027] Martin M (2011) Cutadapt removes adapter sequences from high‐throughput sequencing reads. EMBnet J 17: 10–12

[embr202255432-bib-0028] Michels AA , Fraldi A , Li Q , Adamson TE , Bonnet F , Nguyen VT , Sedore SC , Price JP , Price DH , Lania L *et al* (2004) Binding of the 7SK snRNA turns the HEXIM1 protein into a P‐TEFb (CDK9/cyclin T) inhibitor. EMBO J 23: 2608–2619 15201869 10.1038/sj.emboj.7600275 PMC449783

[embr202255432-bib-0029] Muniz L , Egloff S , Kiss T (2013) RNA elements directing *in vivo* assembly of the 7SK/MePCE/Larp7 transcriptional regulatory snRNP. Nucleic Acids Res 41: 4686–4698 23471002 10.1093/nar/gkt159 PMC3632141

[embr202255432-bib-0030] Nguyen VT , Kiss T , Michels AA , Bensaude O (2001) 7SK small nuclear RNA binds to and inhibits the activity of CDK9/cyclin T complexes. Nature 414: 322–325 11713533 10.1038/35104581

[embr202255432-bib-0031] Noe Gonzalez M , Blears D , Svejstrup JQ (2021) Causes and consequences of RNA polymerase II stalling during transcript elongation. Nat Rev Mol Cell Biol 22: 3–21 33208928 10.1038/s41580-020-00308-8

[embr202255432-bib-0032] O'Keeffe B , Fong Y , Chen D , Zhou S , Zhou Q (2000) Requirement for a kinase‐specific chaperone pathway in the production of a Cdk9/cyclin T1 heterodimer responsible for P‐TEFb‐mediated tat stimulation of HIV‐1 transcription. J Biol Chem 275: 279–287 10617616 10.1074/jbc.275.1.279

[embr202255432-bib-0033] Peng J , Zhu Y , Milton JT , Price DH (1998) Identification of multiple cyclin subunits of human P‐TEFb. Genes Dev 12: 755–762 9499409 10.1101/gad.12.5.755 PMC316581

[embr202255432-bib-0034] Picelli S , Faridani OR , Bjorklund AK , Winberg G , Sagasser S , Sandberg R (2014) Full‐length RNA‐seq from single cells using smart‐seq2. Nat Protoc 9: 171–181 24385147 10.1038/nprot.2014.006

[embr202255432-bib-0035] Quaresma AJC , Bugai A , Barboric M (2016) Cracking the control of RNA polymerase II elongation by 7SK snRNP and P‐TEFb. Nucleic Acids Res 44: 7527–7539 27369380 10.1093/nar/gkw585 PMC5027500

[embr202255432-bib-0036] Schindelin J , Arganda‐Carreras I , Frise E , Kaynig V , Longair M , Pietzsch T , Preibisch S , Rueden C , Saalfeld S , Schmid B *et al* (2012) Fiji: an open‐source platform for biological‐image analysis. Nat Methods 9: 676–682 22743772 10.1038/nmeth.2019 PMC3855844

[embr202255432-bib-0037] Studniarek C , Tellier M , Martin PGP , Murphy S , Kiss T , Egloff S (2021) The 7SK/P‐TEFb snRNP controls ultraviolet radiation‐induced transcriptional reprogramming. Cell Rep 35: 108965 33852864 10.1016/j.celrep.2021.108965

[embr202255432-bib-0038] Van Herreweghe E , Egloff S , Goiffon I , Jady BE , Froment C , Monsarrat B , Kiss T (2007) Dynamic remodelling of human 7SK snRNP controls the nuclear level of active P‐TEFb. EMBO J 26: 3570–3580 17611602 10.1038/sj.emboj.7601783 PMC1949012

[embr202255432-bib-0039] Xue Y , Yang Z , Chen R , Zhou Q (2010) A capping‐independent function of MePCE in stabilizing 7SK snRNA and facilitating the assembly of 7SK snRNP. Nucleic Acids Res 38: 360–369 19906723 10.1093/nar/gkp977 PMC2811026

[embr202255432-bib-0040] Yang Z , Zhu Q , Luo K , Zhou Q (2001) The 7SK small nuclear RNA inhibits the CDK9/cyclin T1 kinase to control transcription. Nature 414: 317–322 11713532 10.1038/35104575

[embr202255432-bib-0041] Yang Z , Yik JH , Chen R , He N , Jang MK , Ozato K , Zhou Q (2005) Recruitment of P‐TEFb for stimulation of transcriptional elongation by the bromodomain protein Brd4. Mol Cell 19: 535–545 16109377 10.1016/j.molcel.2005.06.029

[embr202255432-bib-0042] Yik JH , Chen R , Nishimura R , Jennings JL , Link AJ , Zhou Q (2003) Inhibition of P‐TEFb (CDK9/cyclin T) kinase and RNA polymerase II transcription by the coordinated actions of HEXIM1 and 7SK snRNA. Mol Cell 12: 971–982 14580347 10.1016/s1097-2765(03)00388-5

[embr202255432-bib-0043] Zhou K , Zhuang S , Liu F , Chen Y , Li Y , Wang S , Li Y , Wen H , Lin X , Wang J *et al* (2022) Disrupting the Cdk9/cyclin T1 heterodimer of 7SK snRNP for the Brd4 and AFF1/4 guided reconstitution of active P‐TEFb. Nucleic Acids Res 50: 750–762 34935961 10.1093/nar/gkab1228 PMC8789079

