# Supplementary material for: hnRNP R negatively regulates transcription by modulating the association of P‐TEFb with 7SK and BRD4
Source: EMBO Rep. 2022 Jul 20;23(9):e55432. doi: 10.15252/embr.202255432 (PMC9442301; doi:10.15252/embr.202255432)
Supplement: Supplementary file 1 — Appendix [file EMBR-23-e55432-s002.pdf]

hnRNP R negatively regulates transcription by modulating the association of P-TEFb with 7SK and BRD4

Changhe Ji, Chunchu Deng, Katharina Antor, Thorsten Bischler, Cornelius Schneider, Utz Fischer, Michael Sendtner & Michael Briese

Table of Contents:

Appendix Figure S1 - Generation of hnRNP R knockout cells, page 2

Appendix Figure S2 - Expression of selected proteins in hnRNP R knockout cells, page 3

Appendix Figure S3 - Enhanced proliferation of *HNRNPR*-/- cells, page 4

Appendix Figure S4 - Detection of nascent RNA in hnRNP R-deficient cells revealed by 5-ethynyl-uridine (EU) labelling, page 5

Appendix Figure S5 - Rescue of Ser2 phosphorylation of RNA pol II and levels of Cyclin T1 and CDK9 in *HNRNPR*-/- cells by hnRNP R-EGFP expression, page 6

Appendix Figure S6 - Association of CDK9 with HSP70 and 90, page 7

Appendix Figure S7 - hnRNP R and hnRNP A1 do not associate with P-TEFb and HEXIM1, page 8

Appendix Figure S8 - Glycerol gradient sedimentation analysis of CDK9 from *HNRNPR*+/+ and -/- cells, page 9

Appendix Figure S9 - Generation of 7SK knockout cells, page 10

Appendix Figure S10 - RNA-seq quality control, page 11

Appendix Table S1 - Sequences of oligonucleotides for *HNRNPR* prime editing, page 12

Appendix Table S2 - Sequences of oligonucleotides for 7SK prime editing, page 12

Appendix Table S3 - Sequences of oligonucleotides for mRNA quantification, page 12

Appendix Table S4 - Sequences of oligonucleotides for *HNRNPR* overexpression, page 12

Appendix Table S5 - Antibodies, page 13

Appendix Table S6 - Sequences of oligonucleotides for qPCR, page 14

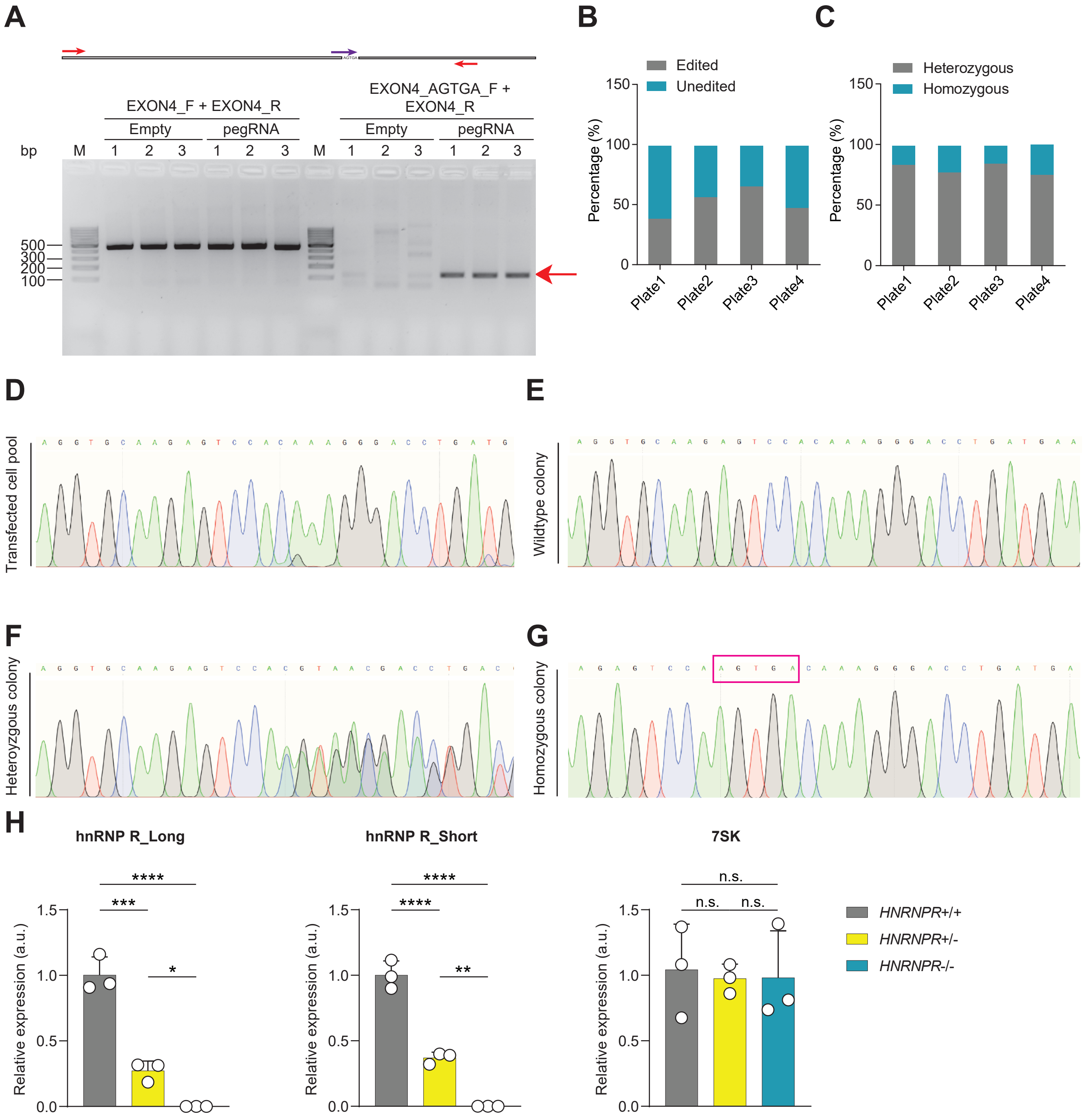

Appendix Figure S1. Generation of hnRNP R knockout cells

A. Agarose gel electrophoresis of the PCR products obtained from control HeLa cells co-transfected with pCMV-PE2 and empty pU6-pegRNA-GG-acceptor, and from HeLa cells co-transfected with pCMV-PE2 and pU6-pegRNA-GG-acceptor harbouring a pegRNA targeting *HNRNPR* exon 4. For PCR, the common primers (red) EXON4_F and EXON4_R annealing upstream and downstream of the prime editing target region, or the primers EXON4_ AGTGA_F (purple), which recognizes the inserted nucleotides, and EXON4_R were used. Thick red arrow indicates the knockout-specific PCR product.

B. Percentage of edited and unedited clonal colonies from four individual 96 well plates.

C. Percentage of heterozygous and homozygous clonal colonies among edited colonies on four individual 96 well plates.

D-G. Sequencing chromatograms from the pool of transfected cells (D), from a *HNRNPR*+/+ wildtype clonal colony (E), from a heterozygous *HNRNPR*+/- clonal colony (F) and from a homozygous *HNRNPR*-/- clonal colony (G). Pink box in (G) indicates inserted sequence.

H. Quantification of relative expression of hnRNP R long and short protein isoforms in Figure 1B, and of 7SK RNA by qPCR. Data are mean with SD; **P* ≤ 0.05, ***P* ≤ 0.01, ****P* ≤ 0.001, *****P* ≤ 0.0001, n.s. not significant; one-way ANOVA with Tukey's multiple comparisons test (n = 3 biological replicates).

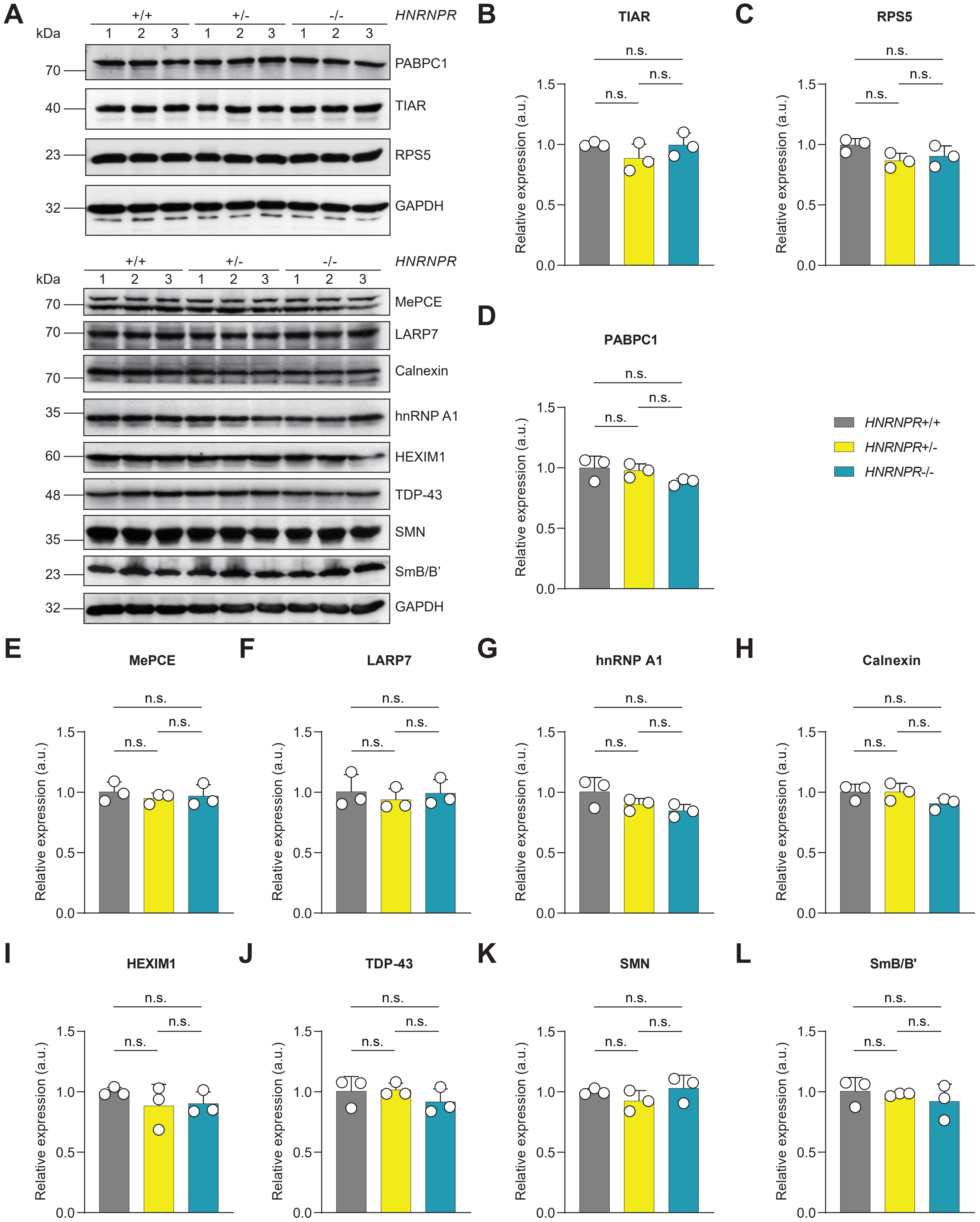

Appendix Figure S2. Expression of selected proteins in hnRNP R knockout cells

A. Western blot analysis of PABPC1, TIAR, RPS5, GAPDH, MePCE, LARP7, Calnexin, hnRNP A1, HEXIM1, TDP-43, SMN, and SmB/B' protein expression in three individual *HNRNPR*+/+, +/- and -/- lines.

B-L. Quantification of relative expression of TIAR (B), RPS5 (C), PABPC1 (D), MePCE (E), LARP7 (F), hnRNP A1 (G), Calnexin (H), HEXIM1 (I), TDP-43 (J), SMN (K) and SmB/B' (L) in (A). Data are mean with SD; n.s. not significant; one-way ANOVA with Tukey's multiple comparisons test (n = 3 biological replicates).

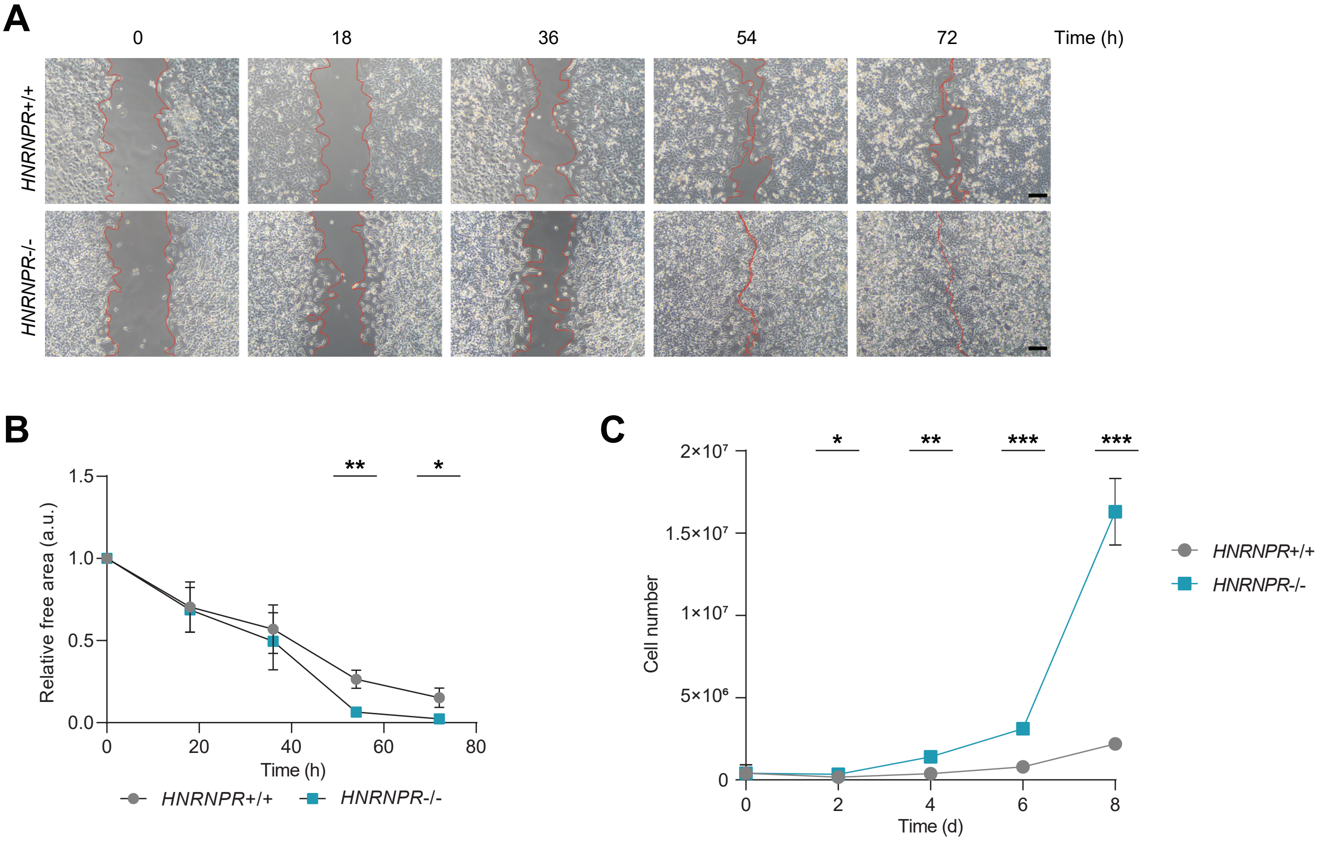

Appendix Figure S3. Enhanced proliferation of *HNRNPR-/-* cells

A. Representative images of wound healing assay at different time points. Scale bar: 120 μm.

B. Quantification of wound closure. Data are mean with SD; $^{*}P \leq 0.05$, $^{**}P \leq 0.01$; unpaired two-tailed *t*-tests (n = 3 biological replicates).

C. Quantification of cell number at different time points. Data are mean with SD; $^{*}P \leq 0.05$, $^{**}P \leq 0.01$, $^{***}P \leq 0.001$; unpaired two-tailed *t*-tests (n = 3 biological replicates).

A

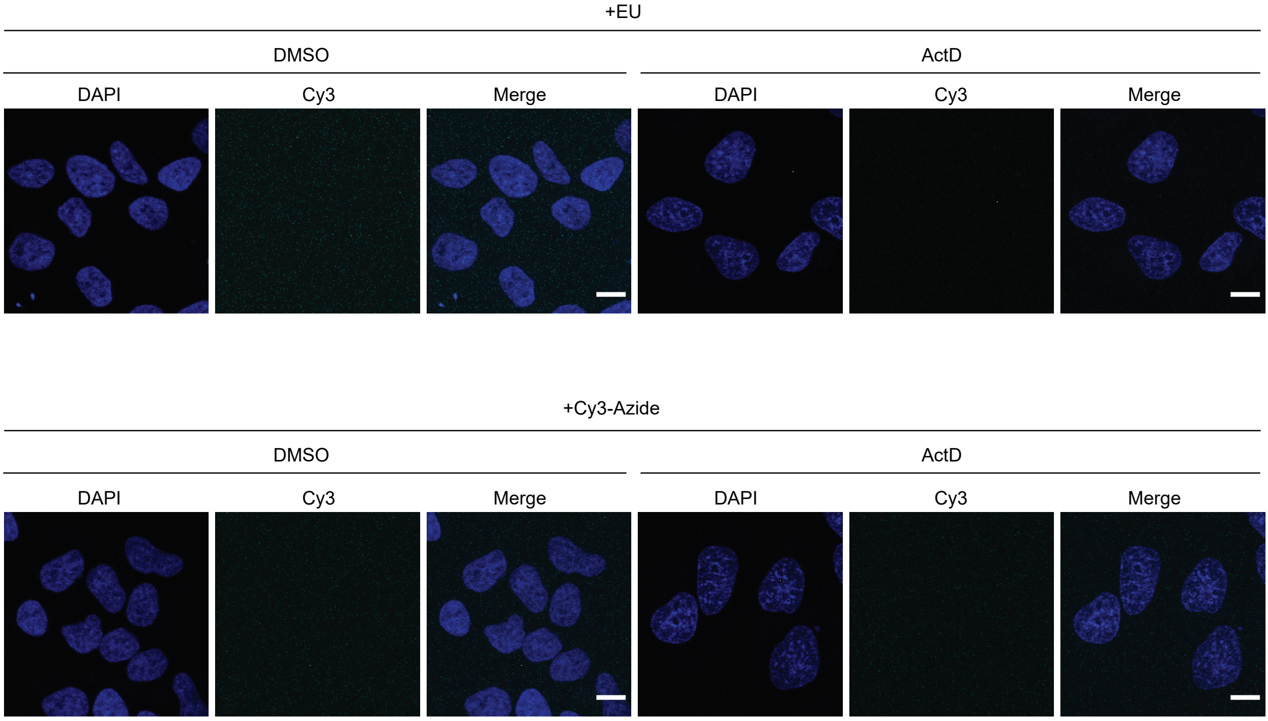

B

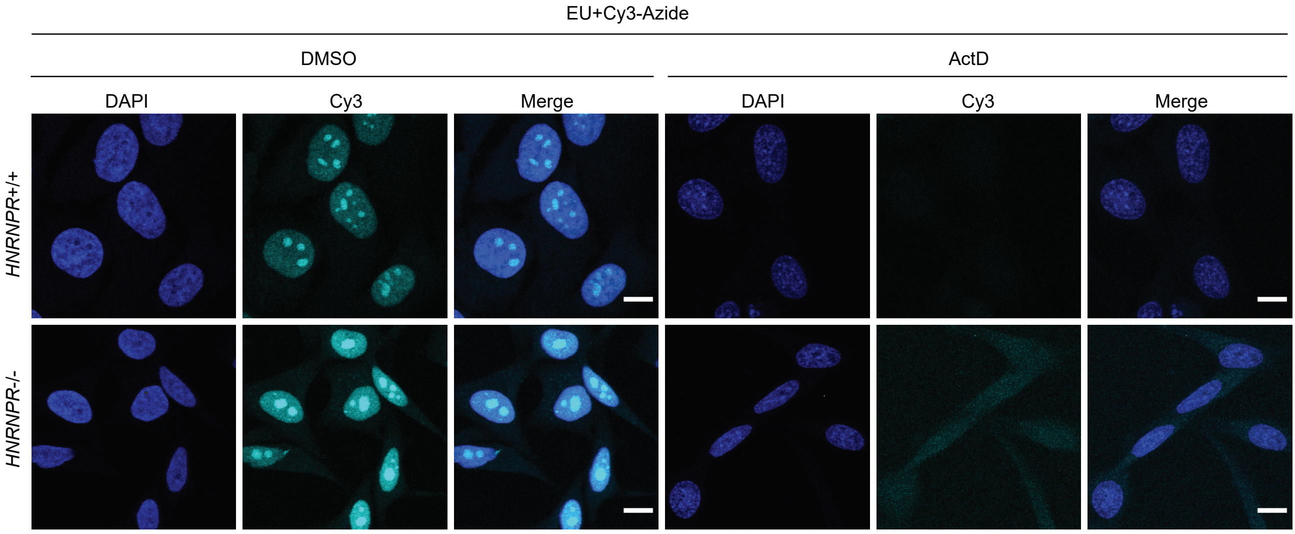

Appendix Figure S4. Detection of nascent RNA in hnRNP R-deficient cells revealed by 5-ethynyl-uridine (EU) labelling

A. Control reactions of HeLa cells exposed to either 5-EU or Cy3-Azide. Cells were treated with DMSO or actinomycin D as indicated. Scale bars: 10 µm.

B. EU labelling of *HNRNPR*+/+ and -/- cells with Cy3-Azide. Cells were treated with DMSO or actinomycin D as indicated. Scale bars: 10 µm.

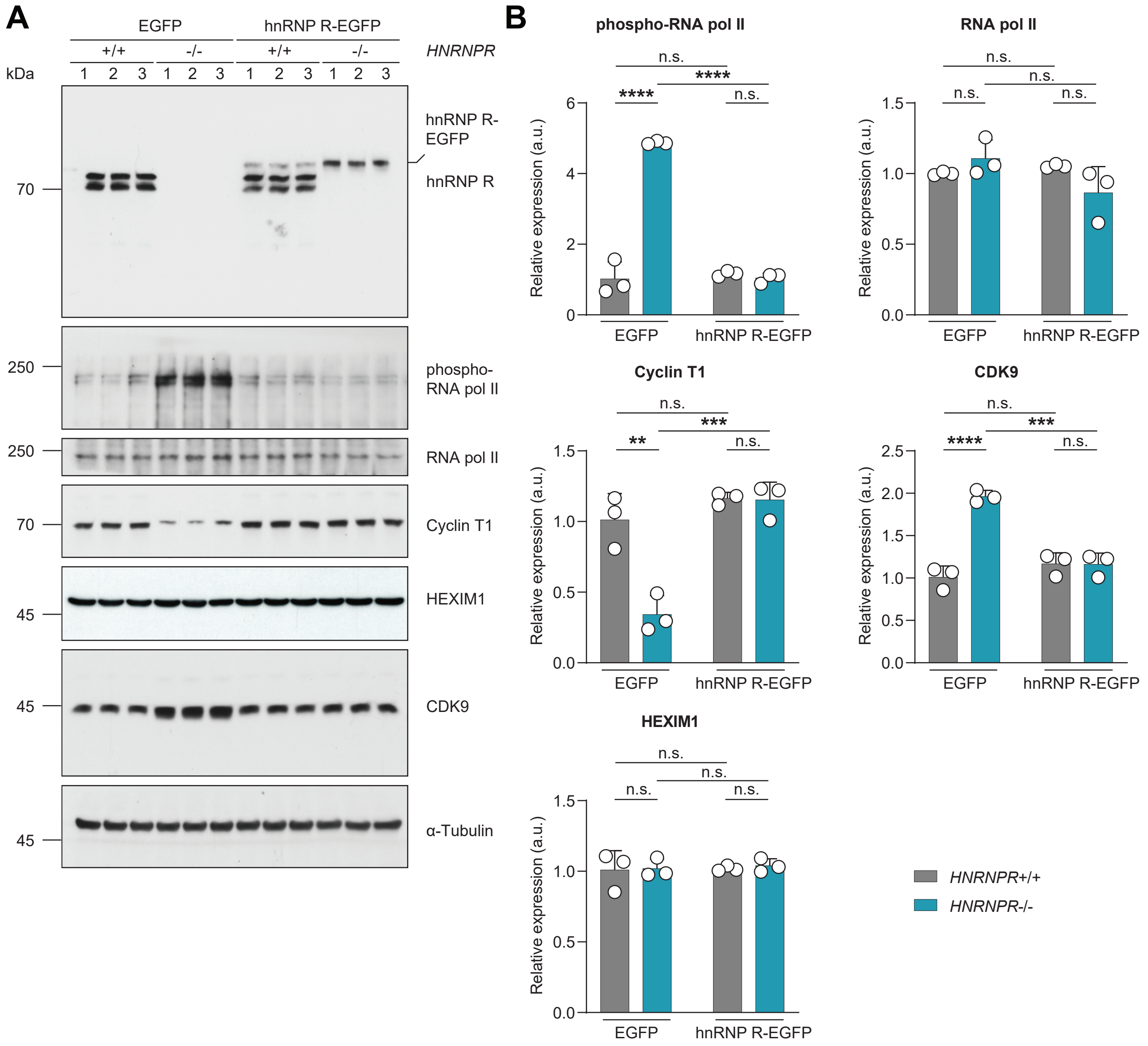

Appendix Figure S5. Rescue of Ser2 phosphorylation of RNA pol II and levels of Cyclin T1 and CDK9 in *HNRNPR*-/- cells by hnRNP R-EGFP expression

A. Western blot analysis of hnRNP R, Ser2-phosphorylated and total RNA pol II, Cyclin T1, HEXIM1, CDK9 and α-Tubulin levels in *HNRNPR*+/+ and -/- cells transfected with pcDNA3-EGFP or pcDNA3-hnRNP R-EGFP.

B. Quantification of protein levels in (A). Data are mean with SD; $^{**}P \leq 0.01$, $^{***}P \leq 0.001$, $^{****}P \leq 0.0001$, n.s. not significant; two-way ANOVA with Sidak's multiple comparisons test (n = 3 biological replicates).

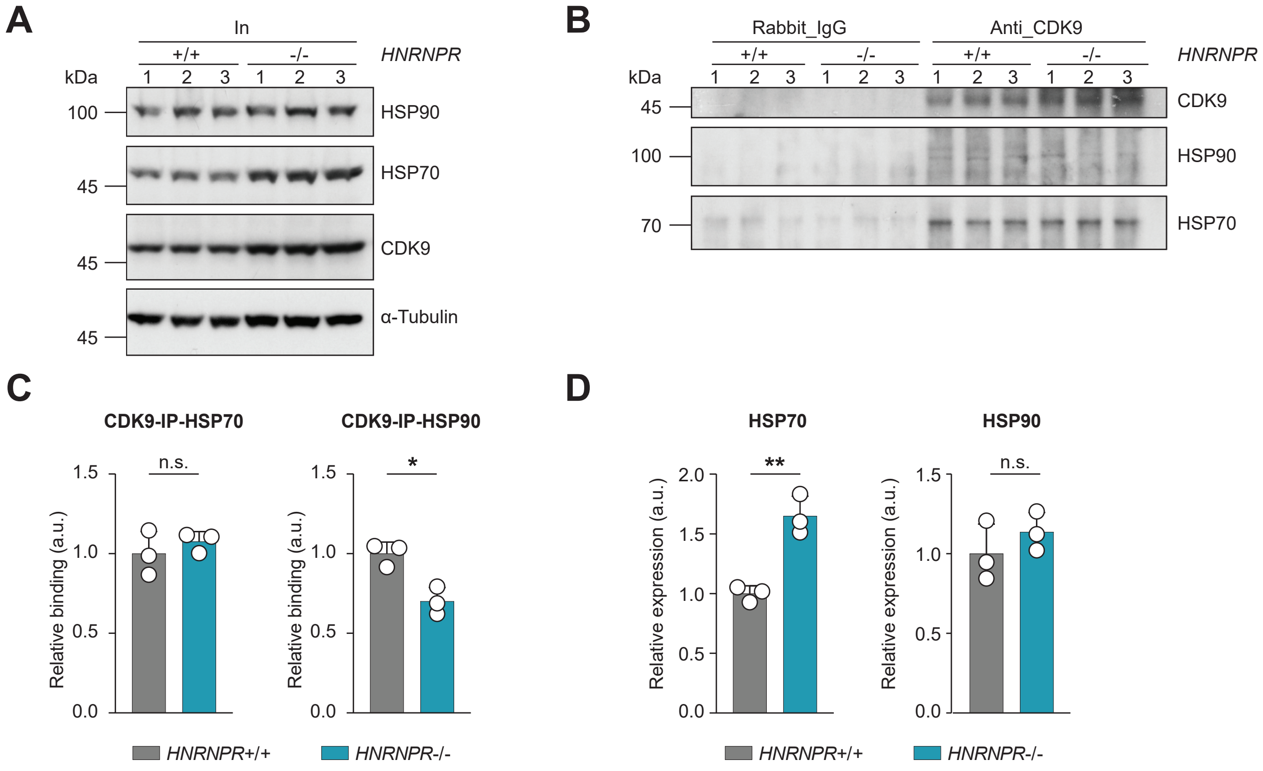

Appendix Figure S6. Association of CDK9 with HSP70 and 90

A. Western blot analysis of HSP90, HSP70, CDK9 and α-Tubulin in the input lysates used for co-immunoprecipitation.

B. Western blot analysis of HSP90 and HSP70 co-immunoprecipitated by an anti-CDK9 antibody. Immunoprecipitation with rabbit-IgG antibody was used as control.

C. Quantification of HSP70 and 90 co-immunoprecipitating with CDK9 in (B). Data are mean with SD; *$P \leq 0.05$; unpaired two-tailed *t*-test (n = 3 biological replicates).

D. Quantification of HSP70 and 90 in the input (A). Data are mean with SD; **$P \leq 0.01$; unpaired two-tailed *t*-test (n = 3 biological replicates).

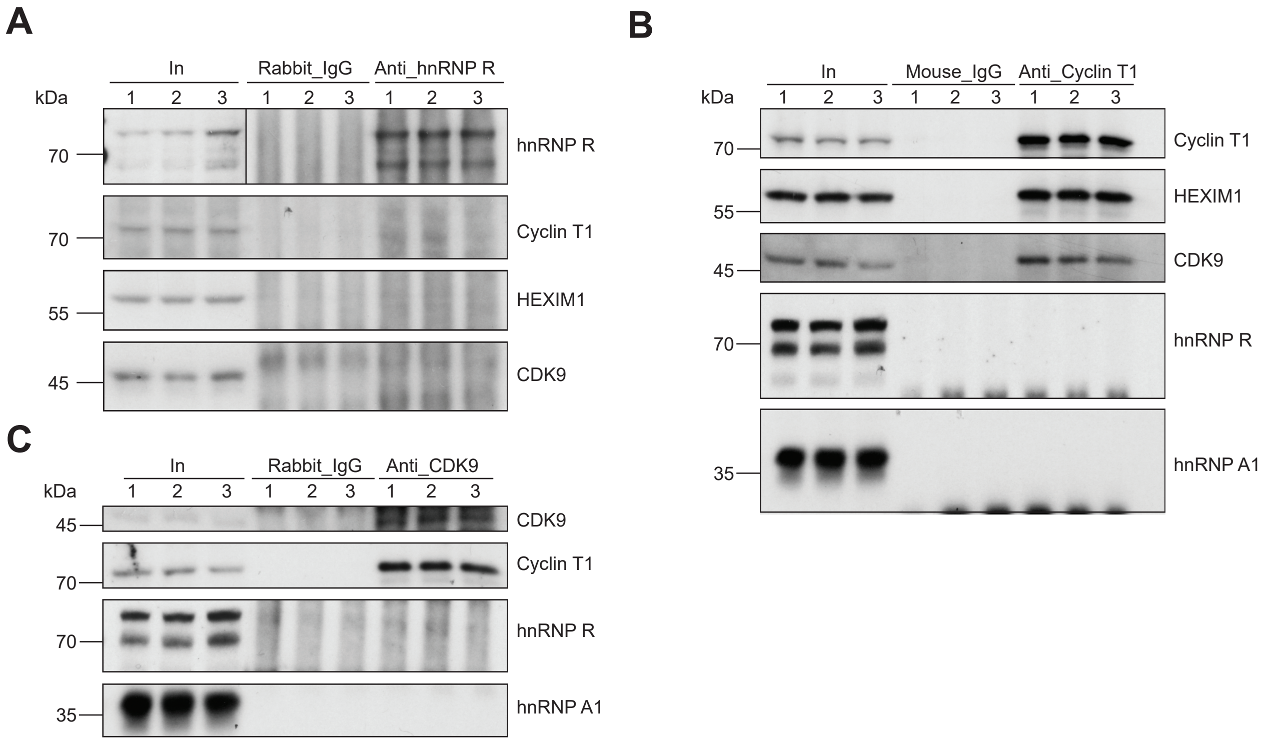

Appendix Figure S7. hnRNP R and hnRNP A1 do not associate with P-TEFb and HEXIM1

A. Western blot analysis of Cyclin T1, HEXIM1 and CDK9 co-immunoprecipitated by an anti-hnRNP R antibody. Immunoprecipitation with rabbit-IgG antibody was used as control.

B. Western blot analysis of HEXIM1, CDK9, hnRNP R and hnRNP A1 co-immunoprecipitated by an anti-Cyclin T1 antibody. Immunoprecipitation with mouse-IgG antibody was used as control.

C. Western blot analysis of Cyclin T1, hnRNP R and hnRNP A1 co-immunoprecipitated by an anti-CDK9 antibody. Immunoprecipitation with mouse-IgG antibody was used as control.

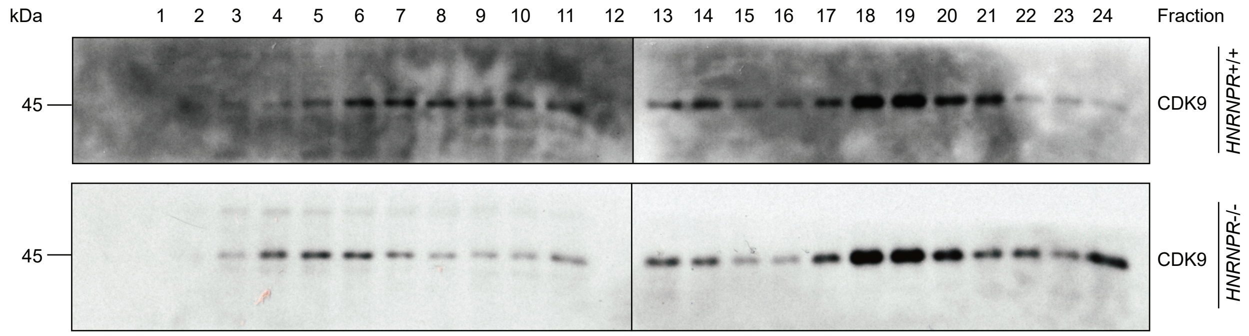

Appendix Figure S8. Glycerol gradient sedimentation analysis of CDK9 from *HNRNPR*+/+ and -/- cells

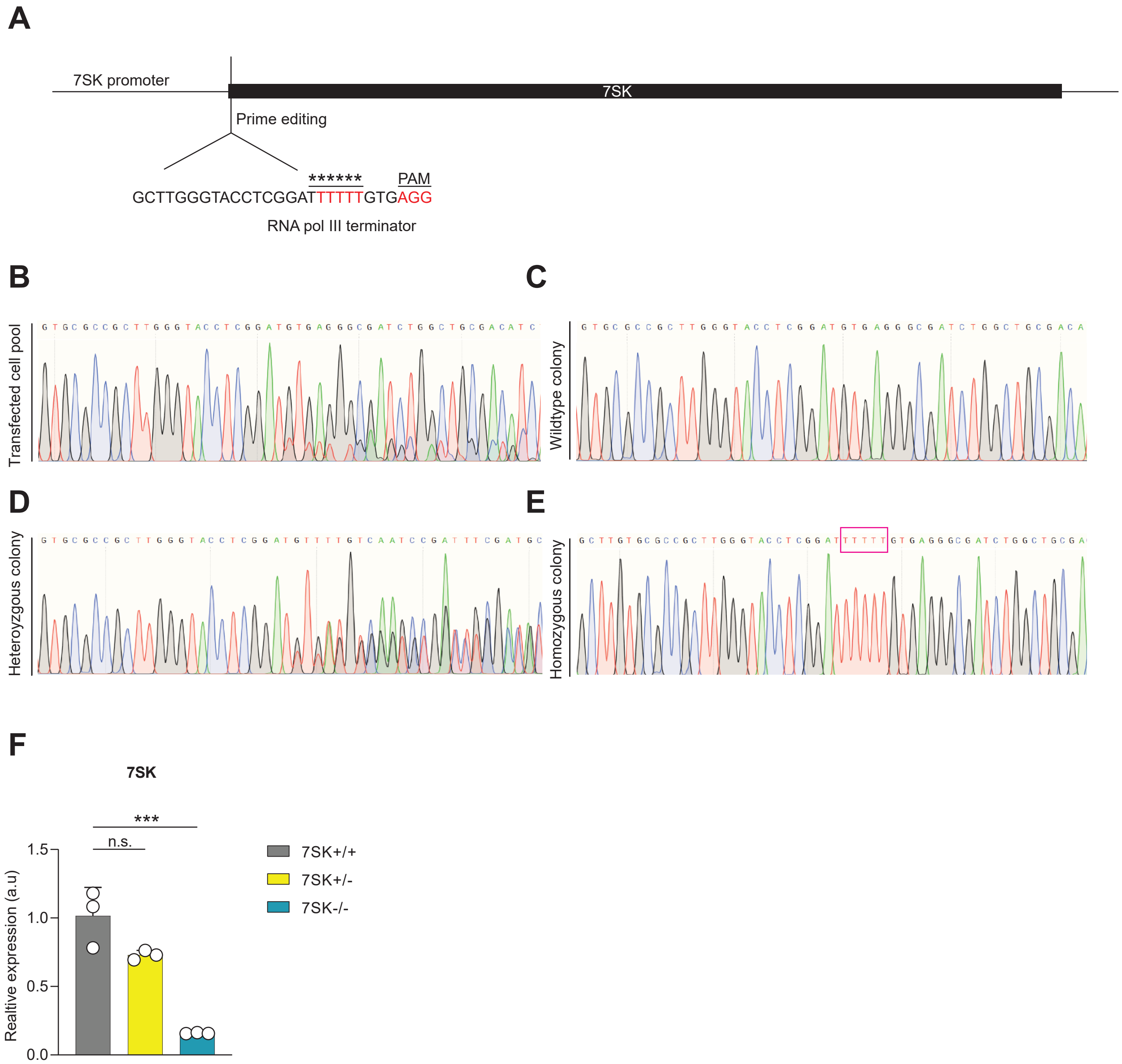

Appendix Figure S9. Generation of 7SK knockout cells

A. Schematic of the strategy for the generation of 7SK knockout cells by prime editing. The T(5) sequence inserted by prime editing is marked in red, the poly(T) sequence acting as transcriptional terminator for RNA polymerase III is indicated by asterisks.

B-E. Sequencing chromatograms from the pool of transfected cells (B), from a 7SK+/+ wildtype clonal colony (C), from a heterozygous 7SK+/- clonal colony (D) and from a homozygous 7SK-/- clonal colony (E). Pink box in (E) indicates inserted sequence.

F. Quantification of relative expression of 7SK RNA by qPCR. Data are mean with SD; ***$P \leq 0.001$, n.s. not significant; one-way ANOVA with Tukey's multiple comparisons test (n = 3 biological replicates).

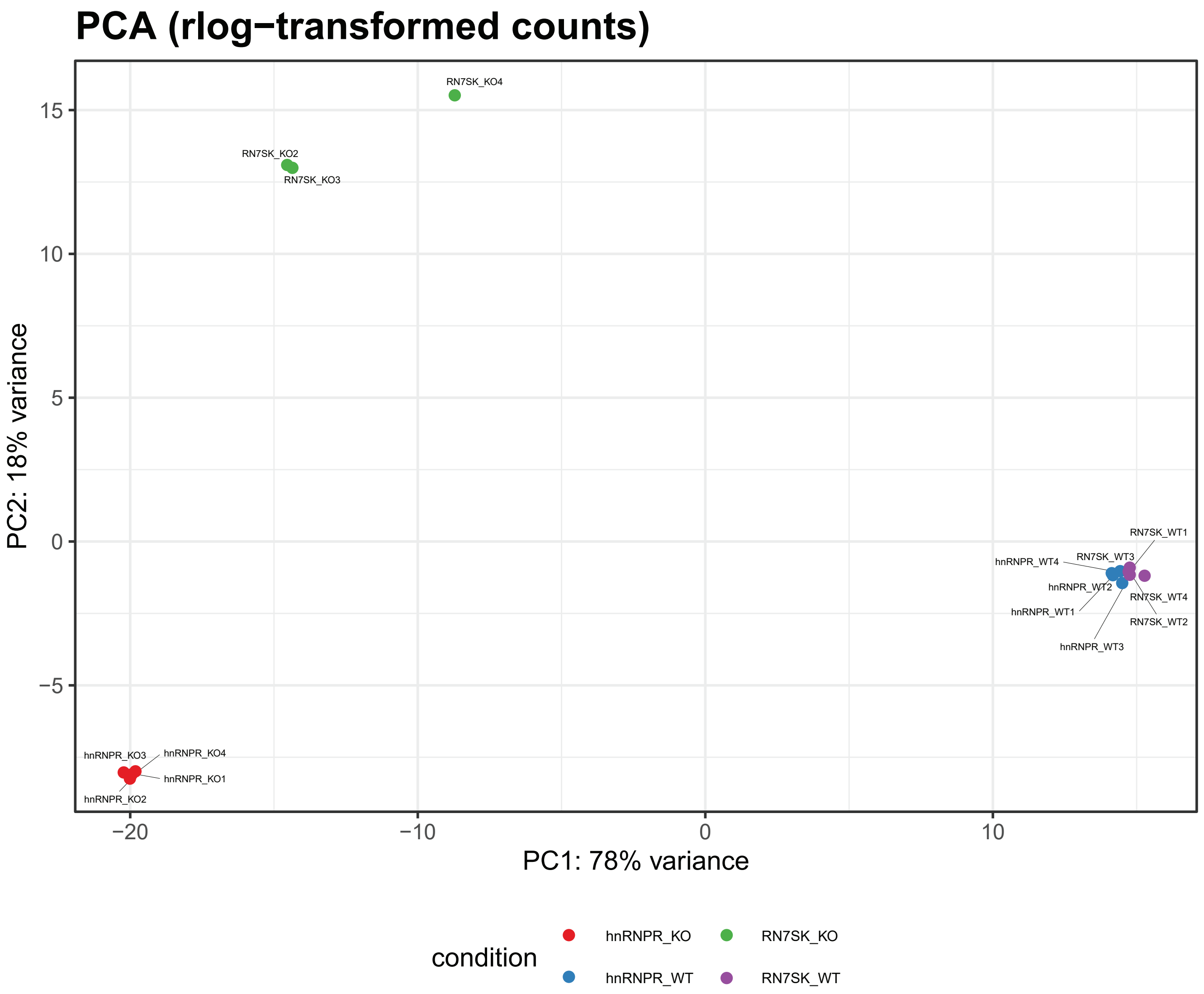

Appendix Figure S10. RNA-seq quality control

PCA plot of RNA-seq data. Each dot represents a sample. Replicates are colour-coded according to genotype.

SUPPLEMENTAL TABLES

Appendix Table S1 - Sequences of oligonucleotides for *HNRNPR* prime editing

Name	Sequence (5'-3')
pegRNA3-1	TAT CTT GTG GAA AGG ACG AAA CAC CGC AAG GTG CAA GAG TCC ACA AGT TTT AGA GCT AGA AAT AGC
pegRNA3-2	GCA CCG ACT CGG TGC CAC TTT TTC AAG TTG ATA ACG GAC TAG CCT TAT TTT AAC TTG CTA TTT CTA GCT CTA AAA C
pegRNA3-3AGTGA	TGA AAA AGT GGC ACC GAG TCG GTG CTC AGG TCC CTT TGT CAC TTG GAC TCT TGC ACC TTT TTT TAA GCT TGG GCC GCT CGA G
EXON4_F	TCCGACATCTGGCAAAGACA
EXON4_AGTGA_F	AGG TGC AAG AGT CCA AGT GA
EXON4_R	GGTCAAATGCCTCTTTCCATGT

Appendix Table S2 - Sequences of oligonucleotides for 7SK prime editing

Name	Sequence (5'-3')
pegRNA3-1	TAT CTT GTG GAA AGG ACG AAA CAC CGG CTT GGG TAC CTC GGA TGT GGT TTT AGA GCT AGA AAT AGC
pegRNA3-2	Same as in Table S1
pegRNA3-3TTTTT	TGA AAA AGT GGC ACC GAG TCG GTG CAG ATC GCC CTC ACA AAA AAT CCG AGG TAC TTT TTT TAA GCT TGG GCC GCT CGA G
7SK_F	AGT ACG ATA AGC AAC TTG ACC T
7SK_TTTTT_F	GCT TGG GTA CCT CGG ATT TTT T
7SK_R	TCTAACTTAGATGGGTAATGGGTCA

Appendix Table S3 - Sequences of oligonucleotides for mRNA quantification

Name	primer (5'–3')	
Oligo-dT30AG	AAG CAG TGG TAT CAA CGC AGA GTA CTT TTT TTT TTT TTT TTT TTT TTT TTT TTT TAG	
TSO	AAG CAG TGG TAT CAA CGC AGA GTA CAT G(+G)(+G)	(+G) is LNA
ISPCR	AAG CAG TGG TAT CAA CGC AGA GT	

Appendix Table S4 - Sequences of oligonucleotides for *HNRNPR* overexpression

Name	Sequence (5'-3')
*HNRNPR*_Long_F	CCA AGC TTG GTA CCG AGC TCG GAT CCG TCG CCA CCA TGG CTA ATC AGG TGA ATG G
*HNRNPR*_R	CGC TAC CGC TAC CGC TGC CGC TAC CCT TCC ACT GTT GCC CAT AAG
*EGFP*_F	CGG CAG CGG TAG CGG TAG CGG CAG CGT GAG CAA GGG CGA GGA GCT
*EGFP*_R	CAC TGG CGG CCG TTA CTA GTG GAT CCC TAC TTG TAC AGC TCG TCC A

Appendix Table S5 - Antibodies

Antibodies	SOURCE	IDENTIFIER
Goat polyclonal anti-HEXIM1	Bio-Rad	Cat#VPA00125
Rabbit polyclonal anti-phospho-RNA Polymerase II (Ser2)	Bethyl Laboratories	Cat#A300-654A; RRID: AB_519341
Mouse monoclonal anti-Pol II (8WG16)	Santa Cruz	Cat# sc-56767; RRID: AB_785522
Rabbit polyclonal anti-LARP7	Proteintech	Cat#17067-1-AP; RRID: AB_2132693
Rabbit polyclonal anti-LARP7	MyBioSource	Cat#MBS9127367
Mouse monoclonal anti-LARP7 (clone E-5)	Santa Cruz	Cat#sc-515209; RRID: AB_2728652
Rabbit polyclonal anti-MePCE	Proteintech	Cat#14917-1-AP; RRID: AB_2250635
Rabbit polyclonal anti-MePCE	Abcam	Cat#ab185991
Rabbit polyclonal anti-hnRNP R	Abcam	Cat#ab30930; RRID: AB_2295532
Rabbit polyclonal anti-hnRNP R (N-term)	Abgent	Cat#AP17239a; RRID: AB_11136203
Rabbit polyclonal anti-Cyclin T1 (D1B6G)	Cell Signaling Technology	Cat# 81464S; RRID: AB_2799973
Mouse monoclonal anti-Cyclin T1 (clone C-6)	Santa Cruz	Cat#sc-271575; RRID: AB_10650141
Rabbit polyclonal anti-Cdk9 (C12F7)	Cell Signaling Technology	Cat#2316S; RRID: AB_2291505
Mouse monoclonal anti-Cdk9 (clone D-7)	Santa Cruz	Cat#sc-13130; RRID: AB_627245
Rabbit monoclonal anti-BRD4 (E2A7X)	Cell Signaling Technology	Cat#13440S; RRID: AB_2687578
Mouse monoclonal anti-hnRNP A1 (clone 4B10)	Santa Cruz	Cat#sc-32301; RRID: AB_627729
Mouse monoclonal anti-GAPDH (clone 6C5)	Calbiochem	Cat#CB1001; RRID: AB_2107426
Goat polyclonal anti-CALNEXIN	SICGEN	Cat#AB0041-200; RRID: AB_2333115
Rabbit polyclonal anti-Histone H3	Abcam	Cat#ab1791; RRID: AB_302613
Mouse monoclonal anti-α-Tubulin (clone B-5-1-2)	Sigma-Aldrich	Cat#T5168; RRID:AB_477579
Mouse monoclonal anti-PABPC1(10E10)	Santa Cruz	Cat#sc-32318; RRID: AB_628097
Mouse monoclonal anti-TIAR	BD Biosciences	Cat#610352; RRID: AB_397742
Mouse monoclonal anti-Ribosomal Protein S5 (A-8)	Santa Cruz	Cat#sc-390935; RRID: AB_2713966
Rabbit polyclonal anti-TDP-43	Proteintech	Cat#10782-2-AP; RRID: AB_615042
Mouse monoclonal anti-SMN (Unconjugated, Clone 8)	BD Biosciences	Cat#610647; RRID: AB_397973
Mouse monoclonal anti-SmB/B'/N (clone 12F5)	Santa Cruz	Cat#sc-130670; RRID: AB_2193856
Rabbit polyclonal Anti-Phospho-CDK9 (Thr186)	Cell Signaling Technology	Cat#2549S; RRID: AB_2077300
Rabbit polyclonal Anti-RNA polymerase II, phospho (Ser2 / Ser9)	Abcam	Cat#ab5095; RRID: AB_304749
Mouse monoclonal Anti-HSP90 (AC88)	Enzo Life Science	Cat#ADI-SPA-830-D; RRID: AB_2039288
Rabbit polyclonal Anti-HSP70	Cell Signaling Technology	Cat#4872S; RRID: AB_2279841

Rabbit polyclonal anti-Cyclin K	Bethyl	Cat#A301-939A, RRID: AB_1547934
Mouse IgG control	Santa Cruz	Cat#sc-2025; RRID: AB_737182
Rabbit IgG control	PeproTech	Cat#500-P00; RRID: AB_2722620
Goat polyclonal anti-Mouse, Peroxidase conjugated	Jackson ImmunoResearch	Cat#115-035-146; RRID: AB_2307392
Donkey polyclonal anti-Rabbit, Peroxidase conjugated	Jackson ImmunoResearch	Cat#711-035-152; RRID: AB_10015282
Donkey polyclonal anti-Goat,Peroxidase conjugated	Jackson ImmunoResearch	Cat#705-035-003; RRID: AB_2340390
Clean-Blot™ IP Detection Reagent (HRP)	Thermo Fisher Scientific	Cat#21230
Donkey polyclonal anti-Mouse, Cy™3 conjugated	Jackson ImmunoResearch	Cat#715-165-151; RRID: AB_2315777
Donkey polyclonal anti-Rabbit, Cy™5 conjugated	Jackson ImmunoResearch	Cat#711-175-152; RRID: AB_2340607

Appendix Table S6 - Sequences of oligonucleotides for qPCR

Gene	Forward primer (5'–3')	Reverse primer (5'–3')
HNRNPR	AAGTCACAGAGGGTTTGG TG	GCTTGTGCTGCTGACTTG TG
7SK	ATTGATCGCCAGGGTTGATTCG	ATGGACCTTGAGAGCTTGTTTG
tRNA	GTCAGGATGGCCGAGCGGTCTAAG	AGGGGAGACTGCGACCTGAA
GAPDH	GCAAATTCCATGGCACC	CGCCAGTGGACTCCACGAC
