# Supplementary figures and images for: hnRNP R negatively regulates transcription by modulating the association of P‐TEFb with 7SK and BRD4
Source: EMBO Rep. 2022 Jul 20;23(9):e55432. doi: 10.15252/embr.202255432 (PMC9442301; doi:10.15252/embr.202255432)

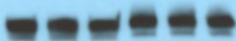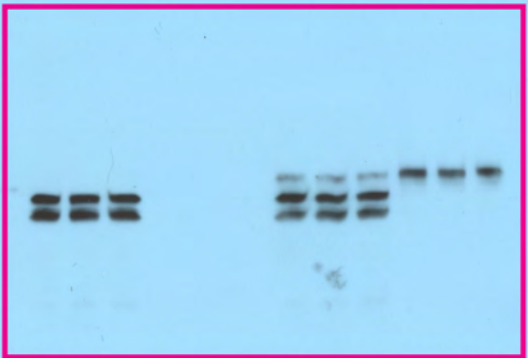

hnRNP R\_EGFP

hnRNP R

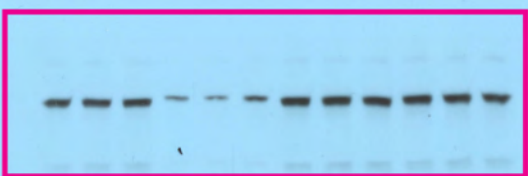

CyclinT1

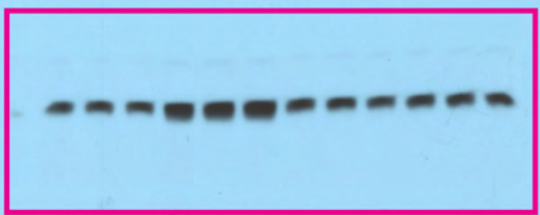

CDK9

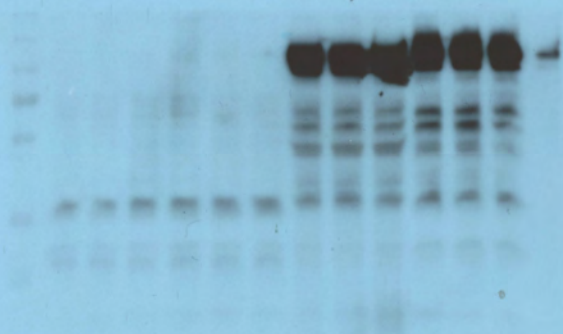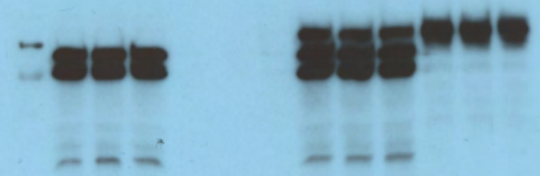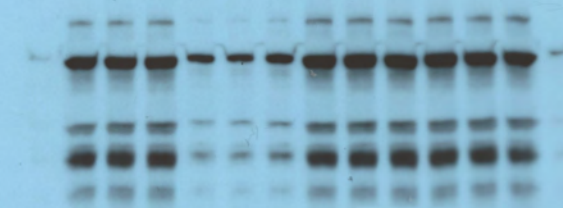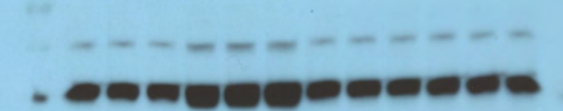

phospho-  
RNA pol II

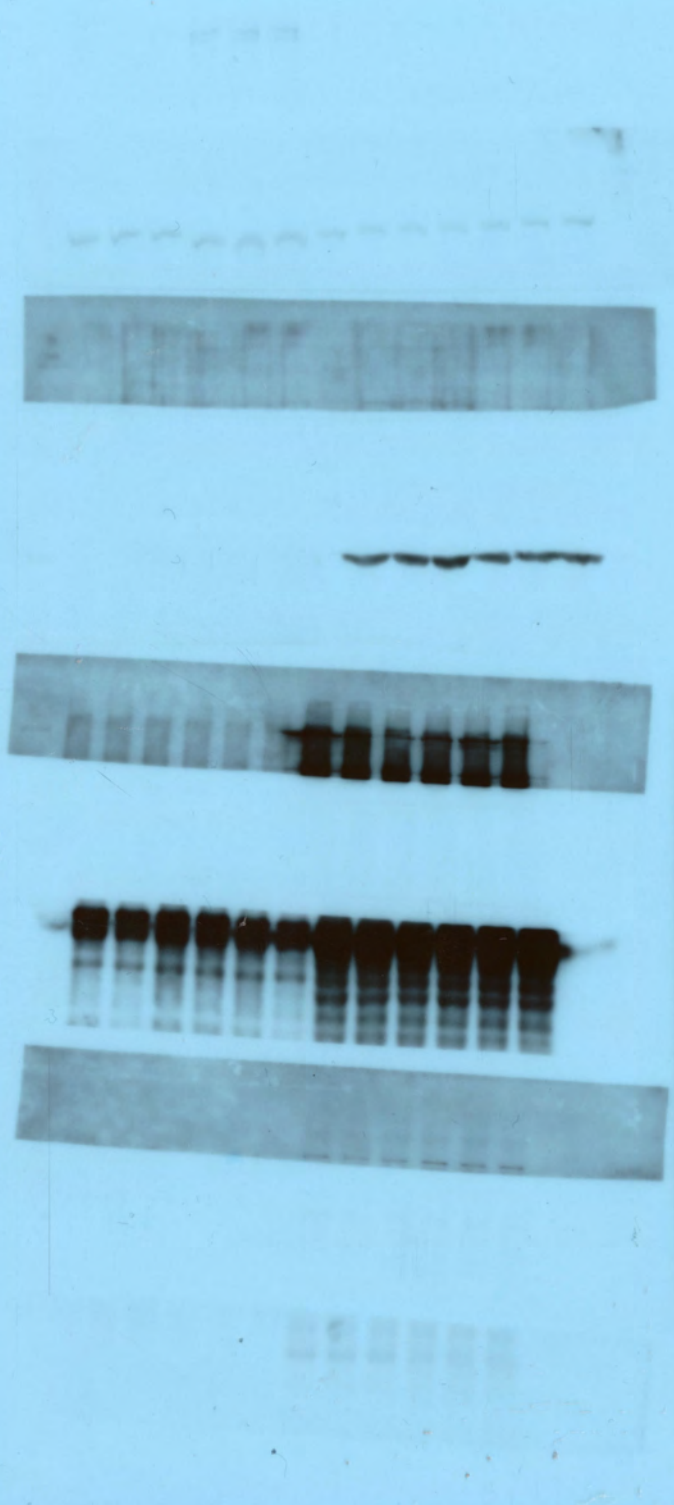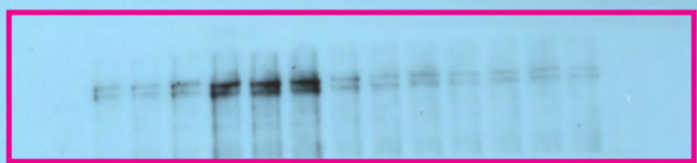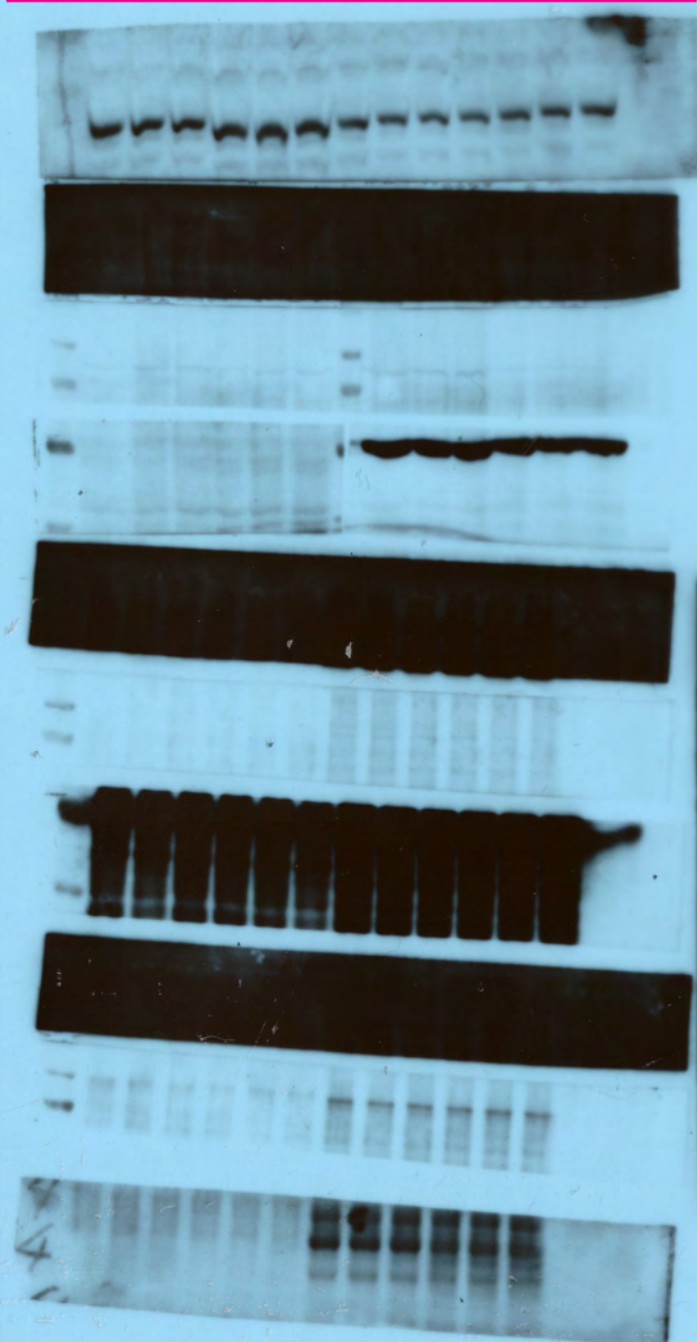

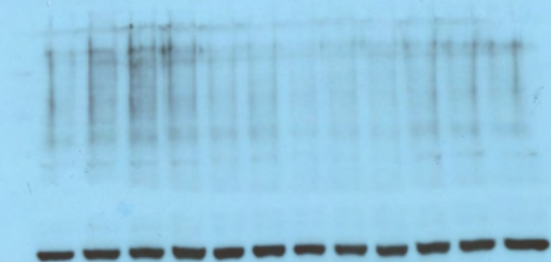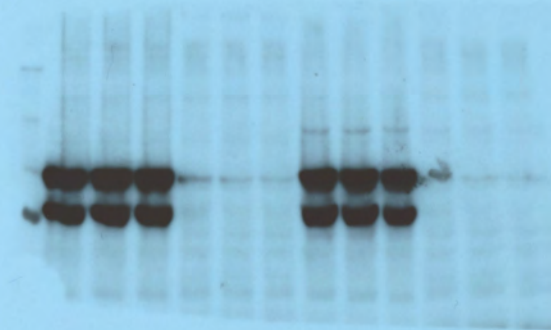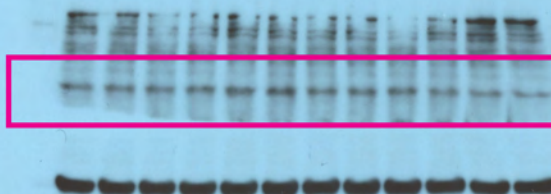

Total\_poll

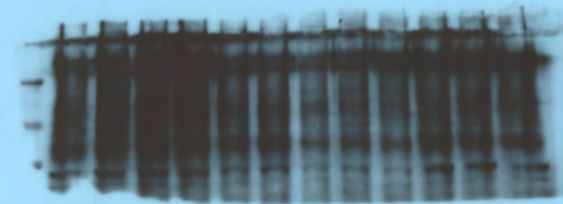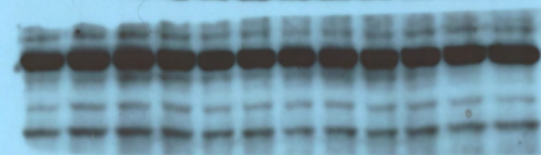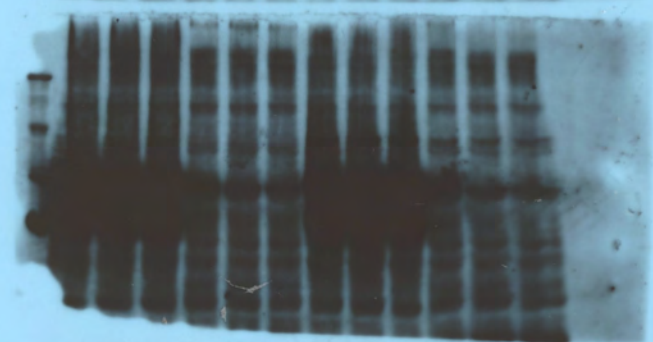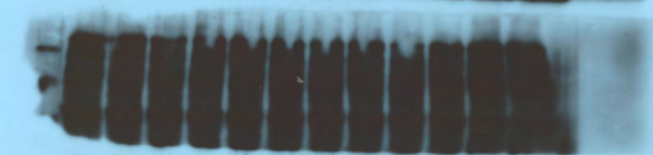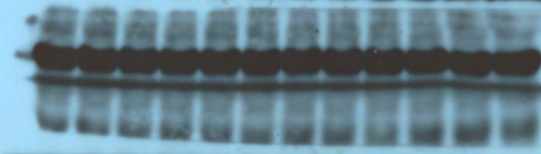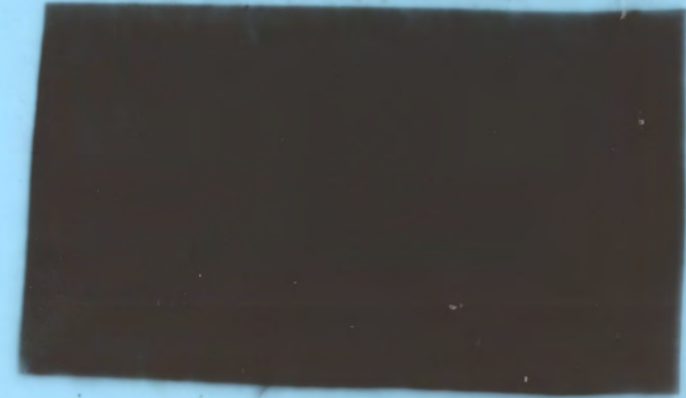

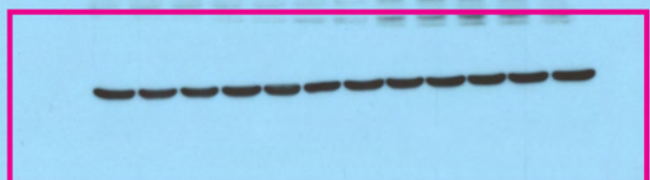

HEXIM1

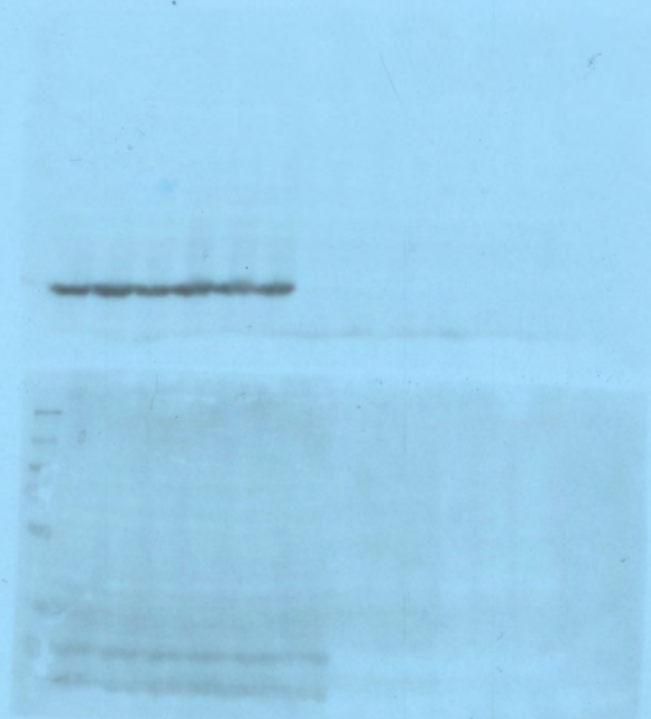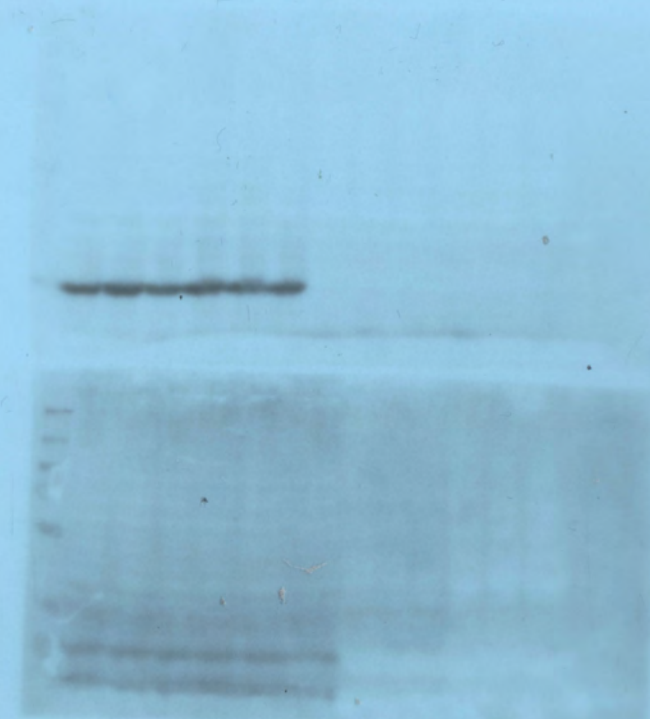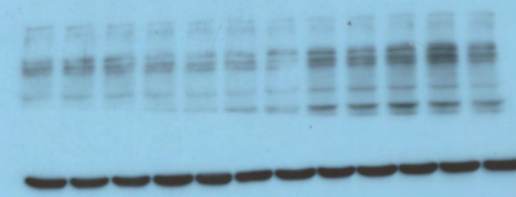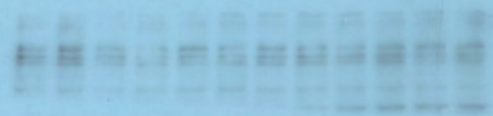

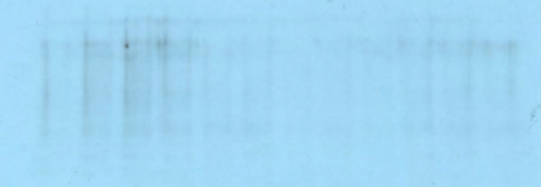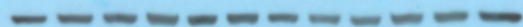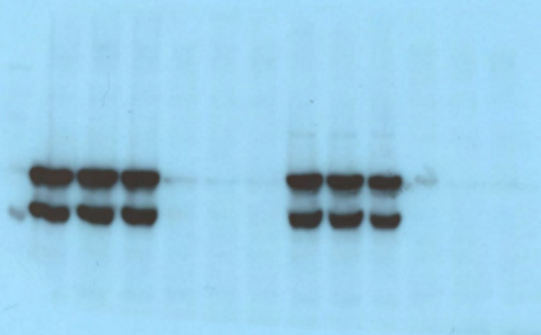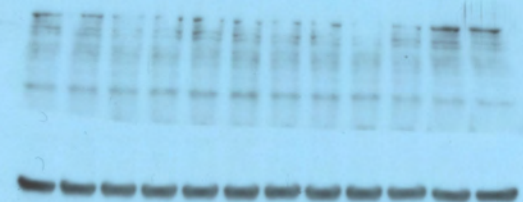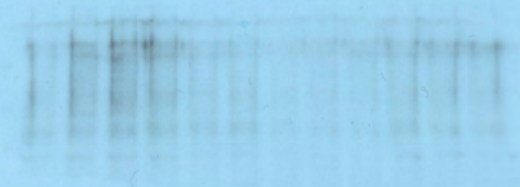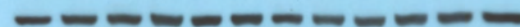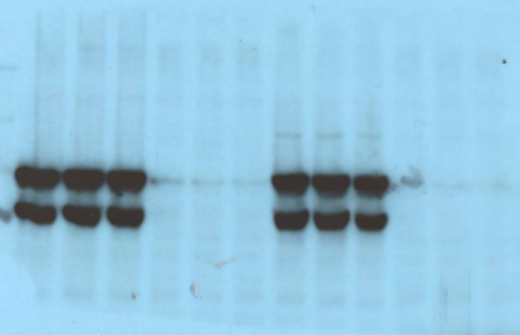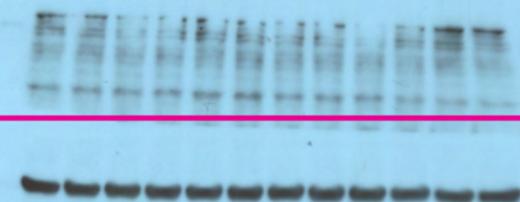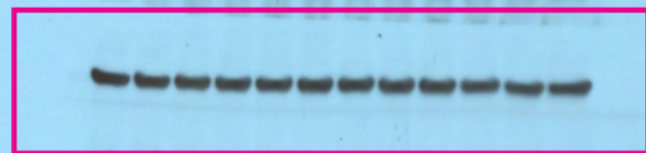

$\alpha$ -Tubulin

Supplement: Supplementary file 5 — Source Data for Appendix [file EMBR-23-e55432-s003.zip › EMBR_2210_Source data Appendix Figure S5A.pdf]

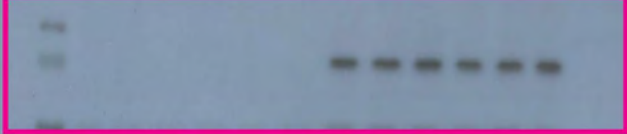

LARP7

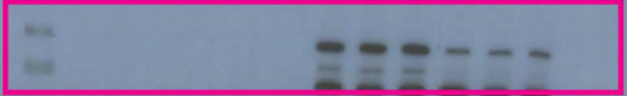

Cyclin T1

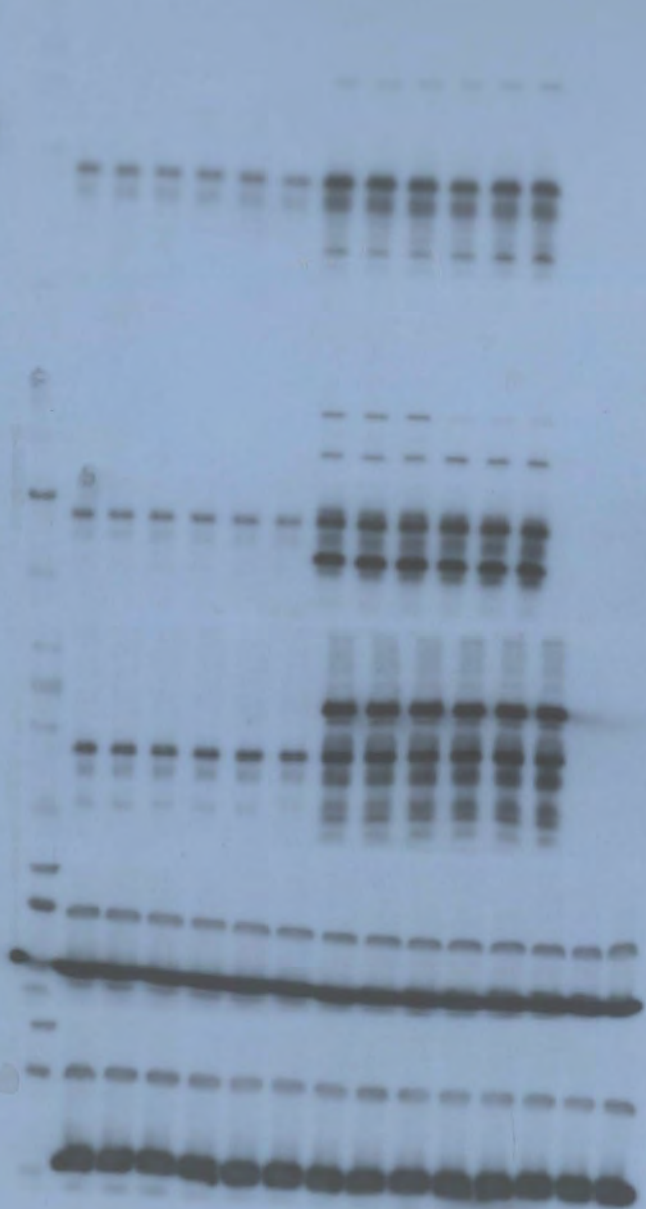

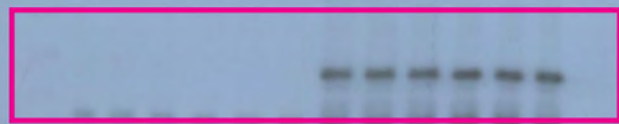

HEXIM1

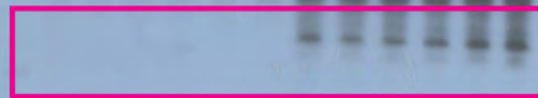

hnRNP A1

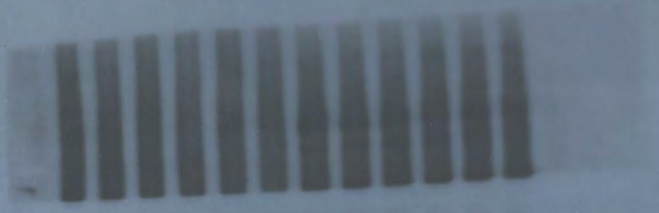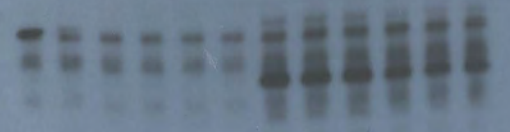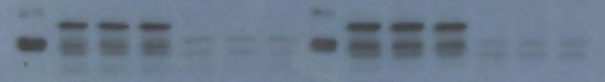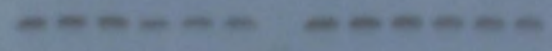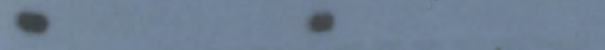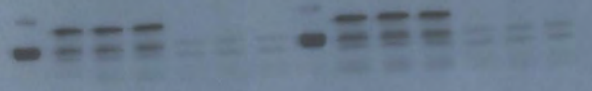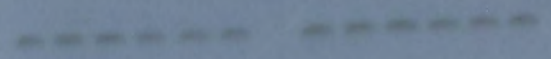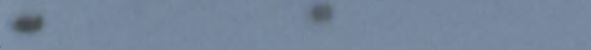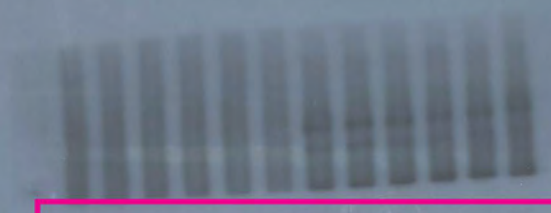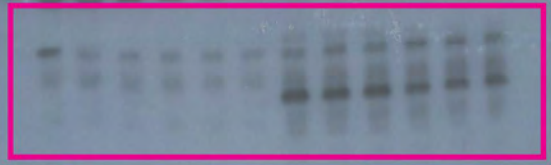

CDK9

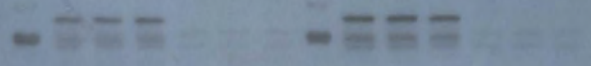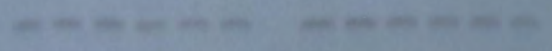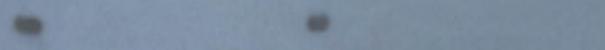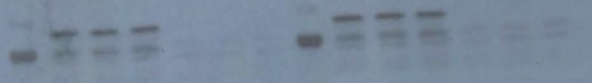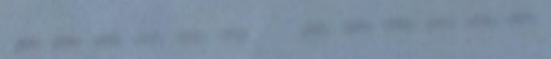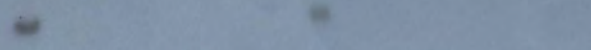

Supplement: Supplementary file 6 — Source Data for Figure 3 [file EMBR-23-e55432-s001.pdf]
